# Identifying Structure and Texture of Metal–Organic Framework Cu_2_(bdc)_2_(dabco) Thin Films by Combining X‑ray Diffraction and Quantum Mechanical Modeling

**DOI:** 10.1021/acs.cgd.4c01433

**Published:** 2025-05-19

**Authors:** Mario Fratschko, Nina Strasser, Narges Taghizade, Mercedes Linares-Moreau, Jan C. Fischer, Tonghan Zhao, Ian A. Howard, Paolo Falcaro, Egbert Zojer, Roland Resel

**Affiliations:** † Institute of Solid State Physics, 27253 Graz University of Technology, Petersgase 16, 8010 Graz, Austria; ‡ Institute of Physical and Theoretical Chemistry, Graz University of Technology, Stremayrgasse 9, 8010 Graz, Austria; § Institute of Microstructure Technology, 150232 Karlsruhe Institute of Technology, Hermann-von Helmholtz-Platz 1, 76344 Eggenstein-Leopoldshafen, Germany

## Abstract

This study describes a strategy for unambiguously determining metal–organic framework (MOF) thin film structures, which is demonstrated for a pillar-layer MOF consisting of Cu paddlewheel nodes connected by benzene-1,4-dicarboxylate (bdc) linkers and 1,4-diazabicyclo[2.2.2]­octane (dabco) pillars. An initial structural model is derived by isostructural replacement from the material’s Zn^2+^ analogue. This is followed by a structure optimization using density functional theory. The model is supported by comparing calculated and measured diffraction patterns and infrared spectra for two differently grown thin films. Key to verifying the structure and identifying the thin film texture are grazing incidence X-ray diffraction (GIXD) experiments with rotating samples. These probe the majority of reciprocal space and thus also allow a straightforward generation of pole figures for various diffraction peaks. Two types of films are prepared either by layer-by-layer deposition or by ceramic-to-MOF conversion. Both share the same phase but display clearly different textures: a uniplanar texture in the case of the layer-by-layer grown film and a distorted axial texture with an epitaxial alignment between MOF and Cu­(OH)_2_ crystallites for the ceramic-to-MOF-converted film. The variations in the texture follow from differences in the substrate surfaces. Our findings highlight the potential of performing GIXD experiments on rotating samples (augmented by theoretical modeling) to (i) determine the texture of MOF thin films and (ii) to solve MOF crystal structures from thin film data even for strongly varying textures.

## Graphical abstract

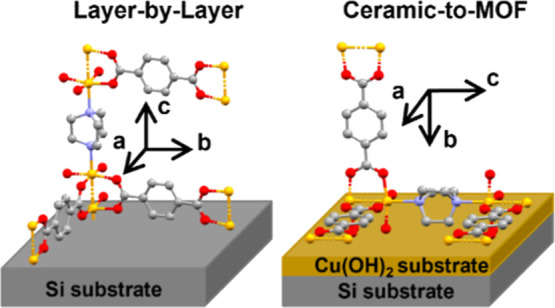

## 1 Introduction

Metal–organic frameworks (MOFs) are extended materials formed by an internal network of metal ions or clusters connected by organic ligands.^1^ They are typically crystalline with high surface areas, tunable pores, and good thermal and chemical stability.^2^ These properties make them ideal for gas storage and separation,^3–6^ drug delivery,^7–10^ sensing^11–13^ and catalysis.^14–16^ The synthesis of MOFs is extremely versatile and can be accomplished by combining metal ions and organic ligands under a variety of conditions, including hydrothermal and solvothermal synthesis, microwave synthesis, or electrochemical and mechanochemical treatments.^17–20^ Usually, MOF crystals with different sizes from tens of nanometers up to millimeters are produced. However, for various applications, such as electronic devices, thin films are required.^21–24^ In order to improve the performance of MOF thin films for different applications, controlling the texture (i.e., orientation) of the crystallites is highly desirable.^25–27^ For example, a defined alignment of the pores can enable enhanced transport of molecules or electrical charge through MOFs, and it can also trigger the alignment of guest-molecules.^28–30^ Oriented MOF thin films are typically fabricated using dedicated techniques, such as gas phase,^31,32^ layer-by-layer (LbL),^27^ or heteroepitaxial^25,30^ growth, among others.

In order to optimize and understand the processes of thin film formation, it is necessary to conduct a detailed crystallographic characterization of the grown films. In this context, X-ray diffraction is the method of choice for obtaining a basic understanding of the type of phase, the preferred orientation of the crystallites relative to the substrate surface, and the epitaxial alignment.^33^ In particular, for thin film characterization, grazing incidence X-ray diffraction (GIXD) is the most suitable technique.^34–36^ Various strategies can be applied to analyze the data, including phase analysis by comparing experimental and calculated peak patterns^37^ or a detailed analysis of peak positions and peak intensities to determine the crystallographic lattices and the arrangement of the metal atoms and linkers within the unit cell.^38^ Of particular interest in this context are GIXD experiments in which the sample is rotated during the measurements (rotating-GIXD) so that a significant fraction of reciprocal space is probed. This provides a much more in-depth knowledge of the actual structure of the studied thin films compared to, e.g., single in-plane and out-of-plane diffraction experiments or GIXD measurements at fixed sample orientations. Still, the latter two techniques are commonly applied for studying MOF thin films due to their faster and easier measurement process, despite the limited amount of data they provide compared with rotating-GIXD experiments. In fact, when combining in-plane and out-of-plane diffraction experiments, one probes only a tiny fraction of reciprocal space. Moreover, the data from GIXD experiments at fixed sample rotation are comprehensive only if one can safely assume a uniplanar texture or randomly oriented crystallites.

Rotating the sample during GIXD measurement besides a more comprehensive structural characterization also allows the extraction of pole figures.^39^ These provide direct information on the distribution of the orientations of crystallites (i.e., the crystallographic texture). They also reveal possible epitactic relationships between substrates and the thin films.^39^ Still, to date they have rarely been used for investigating MOF thin films.^38,40,41^

As a reference system for portraying the potential of the rotating-GIXD approach augmented with quantum-mechanical modeling, we chose Cu_2_(bdc)_2_(dabco). The motivation for this choice is 2-fold: (i) the crystal structure of Cu_2_(bdc)_2_(dabco) has not been explicitly solved so far and (ii) Cu_2_(bdc)_2_(dabco) thin films can be grown with fundamentally different textures depending on the used growth method. Both aspects provide a comparably straightforward way of presenting the potential of the proposed combined theoretical and experimental approach.

In fact, Cu_2_(bdc)_2_(dabco) has been suggested to have an M_2_L_2_P structure^42^ (M: metal ion, L: layer linker, and P: pillar linker). This is based on the assumption that Cu_2_(bdc)_2_(dabco) is isostructural to Zn_2_(bdc)_2_(dabco), whose paddlewheel complexes are connected by benzene-1,4-dicarboxylate (bdc) linkers forming 2D layers. These layers are then connected by (dabco) units.^43,44^ This results in a 3D network. The Zn_2_(bdc)_2_(dabco) framework structure is sketched in Figure 1. In its powder form, Cu_2_(bdc)_2_(dabco) MOF was prepared already about 20 years ago by Seki et al.^43,45^ From a practical point of view, Cu_2_(bdc)_2_(dabco) is interesting due to its high surface area exceeding 700 m^2^/g. Thus, it, for example, has a high potential in gas adsorption and storage.^25,45–47^

**Figure 1:**
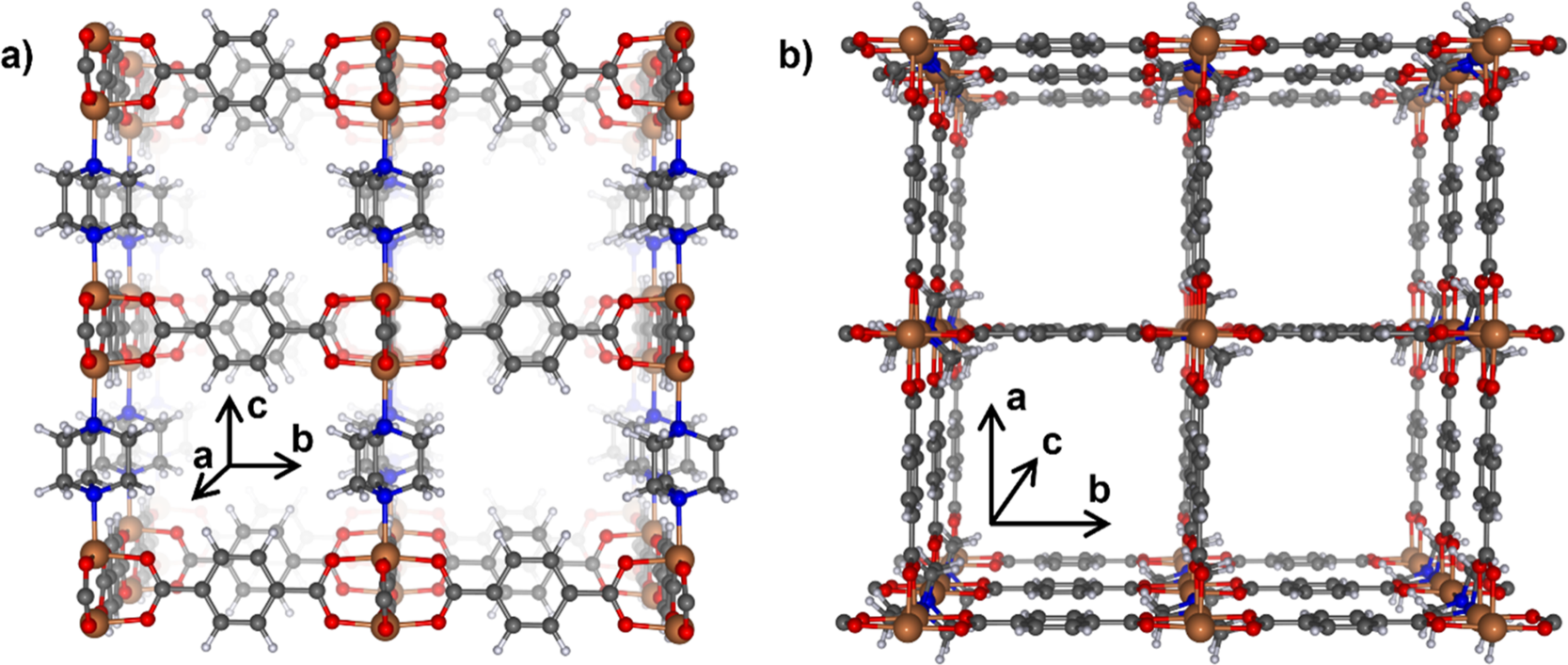
3D-view of Zn_2_(bdc)_2_(dabco) framework structure along (a) the *a*-direction and (b) along the *c*-direction. 2D sheets formed by benzene-1,4-dicaboxylate (bdc) are connected by 1,4-diazabicyclo[2.2.2]­octane (dabco). Zn brown; N blue; O red; C gray; and H white.

For the present study, we focus on the crystallographic characterization of Cu_2_(bdc)_2_(dabco) thin film samples prepared by two complementary methods: (1) LbL growth on silicon^48^ and (2) heteroepitaxial growth by ceramic-to-MOF (CtM) conversion of highly aligned Cu­(OH)_2_ nanobelts which serves as sacrificial material deposited on a silicon substrate.^25^ The direct solution of the crystallographic structure from diffraction experiments on thin films is extremely challenging due to the limited number of available Bragg peaks and due to the difficulty in extracting exact structure factors from the measured intensities.^37,38,49^ Therefore, as a first step, a structural model of the MOF was derived from density functional theory (DFT)-based simulations. The suitability of this crystal structure is then assessed by comparing it to the results of X-ray diffraction experiments. As a complementary approach, measured and simulated IR spectra of the thin films are compared. Based on the suggested crystal structure, as a final step, the texture of the MOF crystallites in the two types of films is determined using pole figures extracted from the rotating-GIXD data. In this way, fundamentally different orientations of the MOFs relative to the substrate surface can be identified for the differently prepared thin films.

## 2 Methods

### 2.1 Experimental Methods

#### 2.1.1 Synthesis

The synthesis of LbL grown Cu_2_(bdc)_2_(dabco) was based on the procedure presented by McCarthy et al.^50^ Thin films were prepared on a cleaned native oxide silicon wafer by two pump sequences alternating between a metal precursor solution (Cu­(CO_2_CH_3_)_2_· H_2_O in ethanol) for 15 min and a linker precursor solution (of 1,4-benzenedicarboxylic acid (H_2_bdc) and dabco in ethanol) for 30 min, including rinsing procedures with ethanol in between. Here, a thin film prepared by 20 cycles is studied. The relevant surface-chemistry is described in ref 50, and there it is also explicitly shown that no functionalization with a surface anchoring molecule on properly cleaned Si substrates is required for obtaining high-quality films.

A second type of sample was prepared by a CtM conversion technique using Cu­(OH)_2_ nanobelts (NBs) as starting points for the MOF growth.^25,51^ The chemical synthesis of the NBs is described in the Supporting Information. The crystalline NBs were injected onto a water bath with a syringe, creating an aligned and uniform film.^52^ After the deposition, a clean silicon substrate is used for an uptake of the NBs, followed by cleaning with ethanol and drying with nitrogen. The substrate covered by the NBs is subsequently immersed for 1 h at 70 °C in 10 mL of methanol-linker solution containing 6.64 mg of H_2_bdc and 287.1 mg of dabco.^25,52,53^ Additionally, a thin film of Cu-bdc (without dabco apical linkers) was grown by an analogous CtM approach, as a reference for identifying certain features in the IR spectra (see below).^25^ All samples were stored and investigated in air under ambient conditions. More details are given in the Supporting Information, Section S1.

#### 2.1.2 Infrared Spectroscopy

For verifying the structure and composition of the Cu_2_(bdc)_2_(dabco) films, Fourier transform infrared (FTIR) spectra were recorded using a Bruker ALPHA spectrometer. Measurements in transmission mode were performed under environmental conditions using 64 scans with a resolution of 4 cm^–1^ in the range between 4000 cm^–1^ and 400 cm^–1^. Data analysis and processing were done using the *OPUS* software (version: 8.5).^54^ All displayed IR spectra (*I*_tm_) are baseline-corrected by dividing the measured intensity (*I*_raw_) by the baseline intensity (*I*_bl_) (determined with OriginPRO 2021b software^55^): $I_{tm}=\frac{I_{raw}}{I_{bl}}$ . The data are presented as absorption spectra (*I*_ab_) by applying the negative logarithm of the corrected data: *I*_ab_ = −log­(*I*_tm_).

#### 2.1.3 GIXD with Rotating Substrate

GIXD experiments were performed at beamline XRD1, Elettra, Trieste. The wavelength of the primary X-rays was 1.40 Å, and a Pilatus 2 M detector at a distance of 200 mm from the sample was used to detect the diffracted beam.

A schematic of the experimental geometry is provided in Figure 2. Two angles define the direction of the primary beam relative to that of the sample. The angle of incidence α_i_ determines the penetration depth and controls the footprint of the primary beam at the sample surface. The incident angles were chosen above the critical angle of Cu_2_(bdc)_2_(dabco) to ensure full penetration into the MOF thin film. Incident angles of 0.2° and of 0.4° were chosen for the LbL and CtM samples, respectively. The azimuthal direction of the primary beam relative to the substrate is defined by the in-plane angle θ_i_ defined around an axis perpendicular to the substrate surface (*z*-axis). The samples were continuously rotated by an overall angle of 360° in 3600 s. The data measured during 10 s were integrated such that we obtained in total 360 2D diffractograms, each covering a range of 1° of θ_i_. The integration interval determines the resolution of the diffraction patterns, and the rotation speed is chosen to provide a sufficiently large signal-to-noise ratio. The advantage of the continuous rotation is that it allows the detection of even very sharp diffraction features, e.g., diffraction peaks of single crystal substrates. The rotation of the sample provides access to large volumes of reciprocal space allowing a comprehensive assessment of the crystal structure largely independent of the specific orientation of the crystallites within the thin film samples. Thus, the approach pursued here is much more versatile than GIXD investigations with a static sample, which provide complete diffraction information only for samples with uniplanar texture or randomly distributed crystallites.

**Figure 2:**
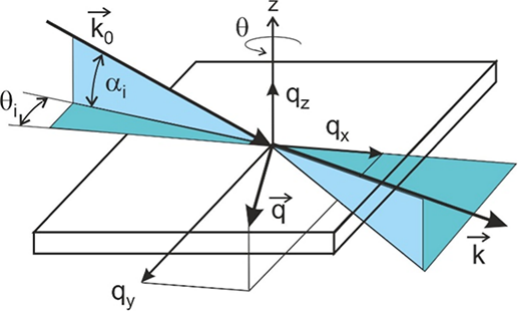
Schematic illustration of the geometry of a GIXD experiment with rotating samples (rotating-GIXD). ${\mathrm{k⃗}}_{0}$ and *k⃗* are the wavevectors of the primary and of the diffracted beam, respectively. The incidence angle α_i_ and the in-plane rotation angle θ_i_ define the direction of the primary beam relative to the sample controlling the penetration depth (α_i_) and the reciprocal space coverage (θ_i_). The rotation axis of the sample is along the *z*-direction, perpendicular to the substrate surface. The scattering vector is obtained by $\mathrm{q⃗}=\mathrm{k⃗}-{\mathrm{k⃗}}_{0}$ . It is used for presenting the diffraction pattern in reciprocal space.

The experimental data were converted to reciprocal space using the software *GIDVis* (version: 2.5), which allows a 3D representation of the diffraction pattern in reciprocal space coordinates.^39^ The reciprocal space maps are plotted as a function of the scattering vector *q* using $q=\frac{4\pi }{\lambda }\sin\theta$ which is separated into an out-of-plane part (*q*_*z*_) and in-plane parts (*q*_*x*_ and *q*_*y*_). The sample coordinate system (*x* and *y*) is selected in a specific direction so that a simple description of the thin film texture is possible: in particular, for CtM samples, we chose the *x*-axis along the preferred long axis direction of the NBs. Reciprocal space images are also presented for a summation of the intensity during a complete sample rotation. In this case, the in-plane part of the scattering vector is plotted as a function of *q*_*xy*_ with *q*_*xy*_^2^ = *q*_*x*_^2^ + *q*_*y*_^2^.

Before analyzing the data, an intensity correction was performed to overcome instabilities of the primary synchrotron beam. For this purpose, a region of defined size was chosen in the diffuse region between the incident beam and the first Bragg peak. The intensity in this region was averaged and considered as the background, which is then removed from the experimental raw data. These corrected data are further processed in *GIDVis* and corrected in terms of solid angle, pixel distance, detector efficiency, polarization factor (for a 95% polarized primary X-ray beam), and Lorentz factor.^56^

The GIXD results are used for analyzing two crystallographic aspects of Cu_2_(bdc)_2_(dabco): in a first step, an identification of the crystal structure is performed, and in a second step, the preferred orientation of the crystals relative to the substrate surface (i.e., the texture) is determined. Qualitative phase analysis is performed by comparing the calculated peak pattern directly with the experimental 2D diffraction pattern in reciprocal space coordinates.

Texture analysis is based on pole figures, which are visualizations of the orientations of the crystals. They are based on the direction of net plane normals (also denoted as poles).^57^ Since pole directions and scattering vectors have identical directions, the connection of diffraction experiments with crystal orientations is possible. A single pole figure gives the intensity distribution of a defined Bragg peak for different directions of scattering vector *q⃗*. The direction of the scattering vector is presented in a circular plot with Euler angles ϕ and ψ, where ϕ denotes the rotation angle around the surface normal of the sample (polar angle) with values in between 0° and 360° and ψ denotes the inclination angle with respect to the surface normal (polar radius) in the range between 0° and 90°.^33^ The intensity (or pole density) at each ϕ/ψ pair is represented by a color code,^58^ with black and yellow representing low and high intensities, respectively.

Pole figures can be computed from the 3D reciprocal space representation of the experimental data. Compared to texture diffractometers, which have to measure individual pole figures for each scattering vector separately, a whole series of pole figures can be computed from rotating-GIXD data. For that, single pole figures are evaluated for a constant value of |*q⃗*|, assuming a specific width of Δ*q* = 0.005 Å^–1^. The experimental pole figures are compared to calculated stereograms (simulated by *Stereopole* (version: 1.2))^59^ based on the crystal lattice under investigation, i.e., based on the lattices of Cu_2_(bdc)_2_(dabco) or Cu­(OH)_2_, respectively. The obtained textures are assigned on the basis of the classification scheme of Heffelfinger and Burton.^60^

### 2.2 Computational Modeling

The geometry of Cu_2_(bdc)_2_(dabco) was optimized by using two complementary codes: the FHI-aims code (version: 210716)^61^ employing atom-centered basis sets and VASP (version: 6.3.0,^62,63^ which combines a plane-wave basis with projector-augmented wave potentials). This was done (i) to show that the obtained results are independent of details of the used methodology and (ii) to utilize specific functionalities readily available in only one of the codes (see below). In these calculations, the Perdew–Burke–Ernzerhof (PBE) functional^64^ was combined with two conceptually different van der Waals corrections: the many-body dispersion correction^65,66^ in the case of FHI-aims and the Grimme's D3 approach when using VASP.^67^ A converged Γ-centered 2 × 2 × 2 k-grid^68^ was employed, and the default “tight” basis sets were used in FHI-aims. In VASP, the same k-grid and a 900 eV energy cutoff were chosen (for convergence tests, see Supporting Information).

As the Cu^2+^ ions contain unpaired spins, ferromagnetic and antiferromagnetic configurations of the Cu-paddlewheels were optimized. Different spin states in the simulations were achieved by initializing the magnetic moments of the Cu ions and by enforcing total multiplicities of the unit cell of S = 1 (open-shell triplet) and S = 0 (open-shell singlet).^69^ For the antiferromagnetic state, the initial magnetic moments of the two copper atoms in each paddlewheel structure were aligned antiparallel. Conversely, for the ferromagnetic state, the copper atoms in the primitive unit cell were initialized, with their spins aligned in parallel. A detailed characterization of the spin states and their impact can be found in Section S2.3 of the Supporting Information. Also, the nonmagnetic (i.e., closed shell singlet) configuration enforcing identical spatial orbitals for the spin-up and spin-down channels was modeled. These simulations reveal an antiferromagnetic ground state of Cu_2_(bdc)_2_(dabco), as described in the Results and Discussion section.

The optimizations started from the structure of Zn_2_(bdc)_2_(dabco) as documented in the Cambridge Crystallographic Data Centre, replacing the Zn^2+^ ions by Cu^2+^ ions.^64^ Subsequently, both the lattice parameters as well as the atomic positions were fully relaxed using the conjugate gradient algorithm in VASP^70^ and an enhanced version of the Broyden–Fletcher–Shanno–Goldfarb (BFGS) algorithm^61^ in FHI-aims until the remaining forces fell below 10^–3^ eV/Å.^61^ Unless specifically stated, all lattice parameters were freely optimized, and no symmetry constraints were imposed (P1 symmetry). In FHI-aims, which straightforwardly allows us to fix unit-cell angles, also a structure with a fully orthogonal unit cell was optimized for the antiferromagnetic configuration.

For calculating vibrational modes with VASP, a finite difference scheme was used employing the Phonopy package,^71^ setting the displacements of individual atoms to 0.01 Å. IR intensities were calculated by using Born effective charges obtained through density functional perturbation theory. The process involves determining the oscillator strengths from the Born effective charges^72^ and atomic displacement vectors. It is commonly observed for MOFs that the resulting PBE-calculated spectra yield a not fully satisfactory description of vibrations. This is in sharp contrast to spectra of molecular crystals.^73,74^ The larger deviations for MOFs are tentatively attributed to the strongly polar nature of their bonds, whose description suffers from the charge over delocalization prevalent in generalized gradient functionals. This can at least in part be mended by the use of hybrid functionals, which have, for example, been successfully applied for modeling the IR spectra of MIL100 and MIL101.^75^ For various materials, especially the B3LYP functional has provided a very good agreement with experimental spectra.^76–79^ Unfortunately we did not succeed in performing hybrid functional calculations for systems as complex as Cu_2_(bdc)_2_(dabco) using VASP due to the high computational costs associated with the plane-wave basis set. Thus, the IR spectra presented in the main manuscript were calculated using the CRYSTAL23 code (version: 1.0.1),^80^ employing the B3LYP^81,82^ functional including Grimmés D3 dispersion correction^67^ with Becke-Johnson damping^83^ (D3/BJ) together with the VTZP^84^ basis set for the linker atoms and the POB-TZVP-rev2 ^85^ basis set for the Zn atoms. The IR spectra discussed in this study were computed based on the fully B3LYP-relaxed structure of Cu_2_(bdc)_2_(dabco) in the antiferromagnetic state. Frequencies were calculated using numerical first-derivatives obtained from the finite difference formula. An anharmonic correction was applied to stretching vibrations that involve hydrogen atoms.^86^ For this, the bond length that involves this type of vibration is varied, and for each distance, the total potential energy is calculated by solving the nuclear Schrödinger equation.^87^ Then, a polynomial of sixth order is fitted to these energy points, allowing one to obtain the anharmonicity constants. Further details of the calculations (including convergence tests) can be found in Section S2.4, where IR spectra for the PBE functional calculated with VASP and CRYSTAL23 are also compared to illustrate the fundamental equivalence of the two approaches.

## 3 Results

### 3.1 Simulated Crystal Structure

As Zn_2_(bdc)_2_(dabco)^64,88^ has a well characterized structure, which is used as a starting point for the simulations, an isostructural conformation for Cu_2_(bdc)_2_(dabco) was assumed. The Zn^2+^ ions were replaced by Cu^2+^ ions, to generate a suitable starting point for the geometry optimizations.^89^ The experiments suggest that Zn_2_(bdc)_2_(dabco) adopts a tetragonal structure (space group *P*4/*mmm* ^64^). In full geometry optimizations, marginal deviations from an orthogonality of the lattice vectors were observed, with deviations amounting to a few tenths of a degree. This applies to Zn_2_(bdc)_2_(dabco) (see Table S2) and to Cu_2_(bdc)_2_(dabco) (see Table 1). The exact magnitude of these marginal deviations from orthogonality depends on the relative spin-orientation in the Cu paddlewheels and also on the details of the applied methodology (see Table 1). Still, the slightly distorted structures are consistently energetically more stable in all simulated systems, but only by ∼0.02 meV/atom. This suggests that the distortions of the unit cells in the optimizations refer to particularly soft degrees of freedom with corresponding vibrations excited already at rather low temperatures such that one can expect the structures to be “orthogonal on average”. Thus, for the following analysis of the experimental GIXD data, an AIMS-calculated structure with a tetragonal unit cell will be used (see last entry of Table 1; for details on its optimization see Methods section).

**Table 1:** Lattice Parameters, Volume, and Energy Differences Δ*E* for Different Magnetic Configurations of Cu_2_(bdc)_2_(dabco) Denoted as Nonmagnetic (NM), Ferromagnetic (FM), and Antiferromagnetic (AFM), Calculated Using FHI-Aims and VASP^a^

	VASP				FHI-aims			
	cell length [Å]	cell angle [°]	volume [Å^3^]	Δ*E* (meV/UC)	cell lengths [Å]	cell angle [°]	volume [Å^3^]	Δ*E* (meV/UC)
NM	*a* = 10.910	α = 89.23	1151.31	193.9	*a* = 10.905	α = 90.97	1146.68	261.3
	*b* = 10.917	β = 90.79			*b* = 10.905	β = 90.93		
	*c* = 9.663	γ = 91.27			*c* = 9.648	γ = 88.10		
FM	*a* = 10.967	α = 89.51	1153.72	60.5	*a* = 10.932	α = 89.87	1154.46	70.3
	*b* = 10.967	β = 90.49			*b* = 10.933	β = 89.88		
	*c* = 9.593	γ = 91.01			*c* = 9.659	γ = 89.41		
AFM	*a* = 10.922	α = 90.58	1156.08	0.0	*a* = 10.919	α = 90.23	1153.92	0.000
	*b* = 10.922	β = 90.56			*b* = 10.919	β = 90.22		
	*c* = 9.692	γ = 89.26			*c* = 9.680	γ = 89.48		
AFM					*a* = 10.925	α = 90.0	1156.98	
					*b* = 10.925	β = 90.0		
					*c* = 9.694	γ = 90.0		

a The last entry in the current table provides the unit-cell parameters for the antiferromagnetic configuration with all cell angles fixed at 90°. As explained in the main text, this structure will be used for the further analysis in the remainder of the manuscript. The abbreviation UC means unit cell.

Regarding the spin states of the paddlewheels in Cu_2_(bdc)_2_(dabco), all calculations attest to an antiferromagnetic (open-shell singlet) ground state. This is also fully consistent with the situation in Cu paddlewheels in HKUST-1 both in experiments and simulations.^69,90–92^ The spin state, however, has only a very minor impact on the unit cell lengths and volumes. For example, in the FHI-aims simulation results reported in Table 1, the unit cell volumes vary by only ∼0.1% between the antiferromagnetic and the ferromagnetic states.

The calculated lattice parameters of Cu_2_(bdc)_2_(dabco) for the fully relaxed AFM ground state configuration are *a* = *b* = 10.919 Å and *c* = 9.680, with α = 90.230°, β = 90.217°, and γ = 89.479°. They essentially do no change in the case of the tetragonal structure (last set of entries in Table 1). The two crystal structures are provided as a crystallographic information file (cif) as part of the Supporting Information. Compared to Zn_2_(bdc)_2_(dabco), the lattice constants of Cu_2_(bdc)_2_(dabco) decreases by 0.1% in both the *a*- and *b*- directions (along the bdc linkers), while the lattice constant increases by 0.8% in the *c* direction (dabco direction) (cf., Table S2).

### 3.2 Confirmation of the Simulated Structure

Two experimental techniques, namely, IR spectroscopy and GIXD, are used to confirm the theoretically determined crystal structure of Cu_2_(bdc)_2_(dabco). The former identifies the chemical building blocks of the MOF by analyzing molecular vibrations, while the latter probes the unit cell parameters as well as the arrangement of the atomic and molecular building blocks within the crystalline lattice. For both methods, the experimental results will be compared to the theoretical predictions based on the crystal structure determined in the previous section.

#### 3.2.1 Infrared Spectroscopy

Figure 3a shows the experimental spectra of both thin film samples and a calculated spectrum of Cu_2_(bdc)_2_(dabco) crystals assuming an isotropic distribution. For the sake of comparison (see below), also the experimental spectrum of Cu-bdc and the simulated spectra of an isolated terephthalic acid molecule (parent compound of bdc linkers) and of the doubly deprotonated dianion are shown. No data for wavenumbers below 1200 cm^–1^ are displayed in Figure 3a, as IR absorption bands associated with the oxidized Si substrates strongly overlap with Cu_2_(bdc)_2_(dabco) features, which prevents an unambiguous assignment of the peaks. The full spectra and a detailed analysis on the peaks below 1200 cm^–1^ are provided in Figure S6. The simulations allow an assignment of experimental peaks to specific crystal vibrations, as shown in Figure 3b. The simulated positions of the most relevant IR-active modes are also provided in Table S5 together with links to the respective animations.

**Figure 3:**
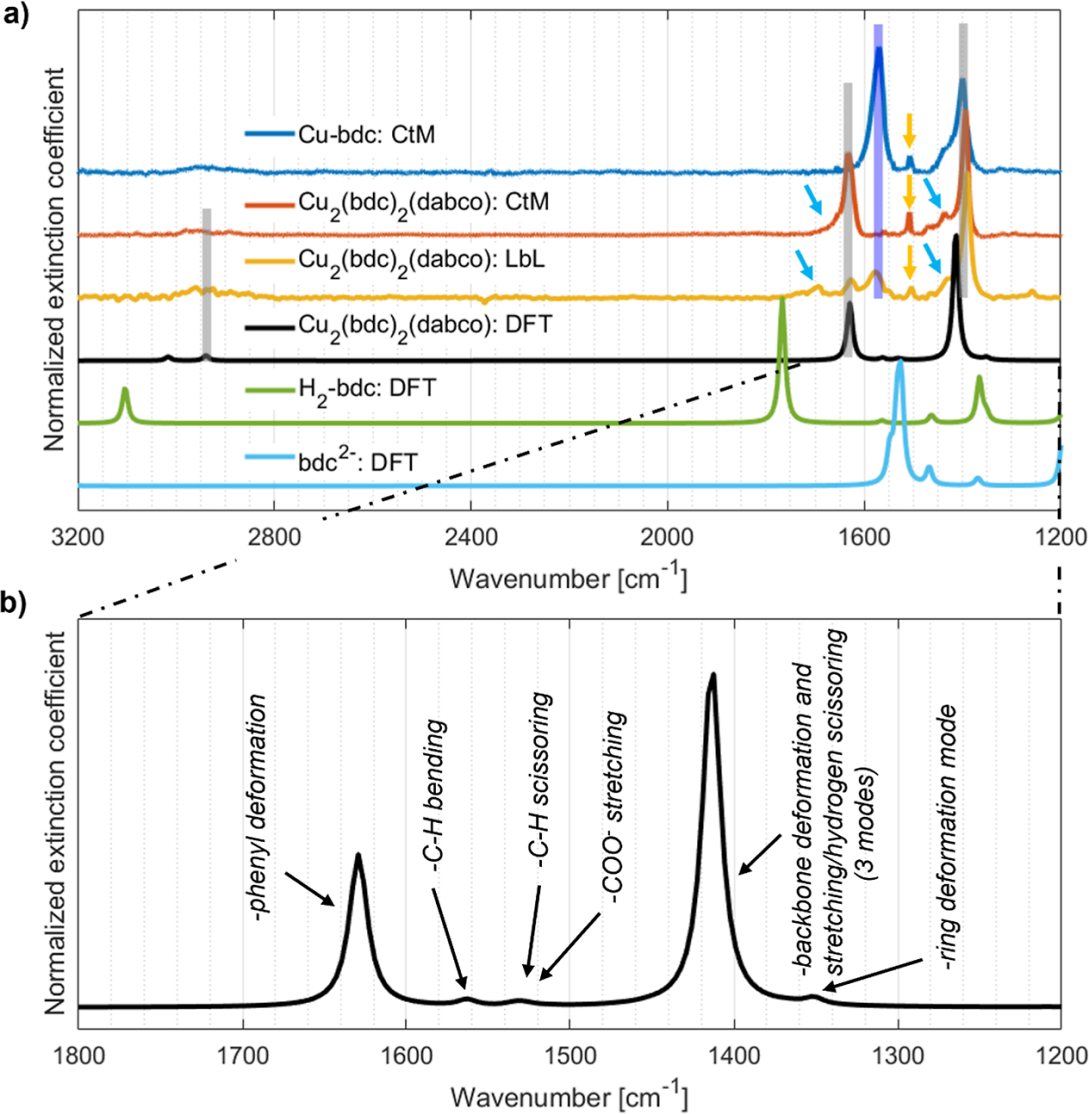
Simulated and measured FTIR spectra of Cu_2_(bdc)_2_(dabco) in the spectral region free of substrate features. Panel (a) compares the experimental result for the ceramic-to-MOF (CtM) sample (red) and for the layer-by-layer (LbL) sample (orange) with simulations on Cu_2_(bdc)_2_(dabco) crystals assuming a random crystal orientation and correcting for anharmonic effects (black). For the sake of comparison, also the measured spectrum of a CtM grown Cu-bdc film (blue line) and the simulated spectra of an isolated terephthalic acid molecule (bdc linker molecule prior to deprotonation; denoted as H_2_(bdc)) and its doubly deprotonated dianion (denoted as bdc^2–^) are shown. The vertical, gray lines highlight the main spectral features of Cu_2_(bdc)_2_(dabco) observed by experiments and in simulations, the vertical blue line denotes the position of the experimental main peak of Cu-bdc, the orange arrows highlight an IR peak associated with bdc dianions (for details see main text), and the cyan arrows denote IR features associated with adsorbates. The Cu-bdc sample shows the same spectrum as reported in ref 25,93 . Panel (b) provides a zoom into the calculated spectrum of the most relevant region of panel (a). It shows positions and IR intensities of active vibrations of the simulated Cu_2_(bdc)_2_(dabco) crystal. Additionally, the nature of the main features are provided (see Table S5).

The two main experimental features in the considered spectral range associated with Cu_2_(bdc)_2_(dabco) are found at 1393 cm^–1^ and 1633 cm^–1^ (thick gray, vertical lines in Figure 3a), consistent with the results of Baumgartner et al.^93^ The experimental peak positions agree well with (i) two essentially degenerate and strongly IR active vibrations simulated for the Cu_2_(bdc)_2_(dabco) crystal (calculated to be at 1414 cm^–1^) and (ii) with a single band simulated to be at 1629 cm^–1^. It has to be mentioned that the calculated positions of the IR active modes depend on the functional used, as discussed in Section 2.2, while the overall shape of the spectra is not seriously affected. Thus, perfect agreement between simulated and measured peak positions cannot be expected. Still, the good correspondence between simulations and experiments is only observed for the calculated IR spectra of the MOF crystal (black line in Figure 3a), while the respective peaks obtained in the simulations of an isolated terephthalic acid molecule (green line in Figure 3a) are strongly shifted to positions at which no peaks are observed in the experiments. This is the first strong indication that Cu_2_(bdc)_2_(dabco) crystals are actually formed in the experiments. In particular, the data show that the terminal groups of the ligands participated in the reactions, leading to thin film formation in a manner consistent with the simulated Cu_2_(bdc)_2_(dabco) crystal structure. The relevance of the IR spectrum of the bdc dianion will be discussed below.

The modes at 1414 cm^–1^ correspond to backbone-stretching vibrations of the bdc linkers accompanied by a scissoring motion of the O–C–O elements, mostly affecting the bonds between the phenylenes and the O–C–O groups. This results in a polarization of the vibrations within the Cu_2_(bdc)_2_(dabco) planes (see labeling in Figure 3b). The line at 1629 cm^–1^ is dominated by an asymmetric deformation vibration in the phenyl unit of the bdc groups, which is in phase for all respective groups in the crystal. The resulting displacements of the central carbon atoms are parallel to the directions of the dabco linkers such that only IR photons polarized in that direction are absorbed. Thus, the observation that the 1629 cm^–1^ peak in the LbL sample (orange line) is much lower than in the CtM sample is a first indication that the LbL sample grows with the dabco linkers preferentially perpendicular to the substrate,^93^ a notion that will later be confirmed by the in-depth analysis of the GIXD data. In the simulations, there are also several weaker IR-allowed vibrations between ∼1300 cm^–1^ and 1700 cm^–1^. Their unambiguous assignment to experimental features is, however, difficult. In contrast, the features found around 3000 cm^–1^ in the simulations can be correlated with the rather broad and weak feature found in the experiments at 2940 cm^–1^. They correspond to C–H stretching vibrations (which are strongly affected by anharmonic effects, see Methods section).

Another prominent feature is the peak found in the LbL spectra at 1577 cm^–1^ (see vertical blue line), which is not observed in the CtM sample. It also does not have an analogue in the simulated spectrum, as the weak simulated features at 1563 cm^–1^ are not intense enough to explain the experimental observations. Thus, we compared the experimental IR spectra of Cu_2_(bdc)_2_(dabco) to that measured for a Cu-bdc reference sample (shown as the topmost line in Figure 3a; for details, see Methods section). Indeed, the most prominent band in Cu-bdc overlaps with the so far unexplained feature in the spectrum of the LbL prepared Cu_2_(bdc)_2_(dabco). This implies that in the thin film grown by the LbL technique Cu-bdc crystals are also formed, which do not contain any dabco pillar linkers. Another feature not occurring in the simulated Cu_2_(bdc)_2_(dabco) spectrum but in all samples containing bdc-linked MOFs is a sharp peak at 1507 cm^–1^ (orange arrow). This feature has also been observed in Baumgartner et al.^93^ in all studied bdc-containing MOFs and it has been associated with the “skeletal ring vibration” of bdc. Indeed, we also find a prominent feature in the respective wavenumber range (peaking at 1526 cm^–1^), when calculating an isolated bdc^2–^ dianion (see the light blue curve in Figure 3a). This suggests that all MOF samples contain deprotonated bdc molecules that are not part of the crystalline Cu_2_(bdc)_2_(dabco) regions and, thus, largely preserve their molecular character, being only loosely associated with (presumably Cu^2+^) counterions to ensure charge neutrality. The shift of around 20 cm^–1^ between the measured and simulated peak positions can be attributed either to the loose interactions with counterions or to the strong methodology-dependence of the simulated peak positions mentioned above.

An unambiguous assignment of the additional peaks and shoulders denoted by the cyan arrows was not possible. In this context, it is, however, worthwhile mentioning that water vapor is known to have vibration–rotation bands in the regions of the cyan arrows. This could be an indication of residual water being present in the samples, potentially in the pores. The water in the pores would, however, need to be extremely weakly bonded such that vibration–rotation bands would occur, as in experiments on water vapors. In passing, it is noted that the IR signatures of vapors of the solvent molecules used in the synthesis (methanol and ethanol) were not observed.

Overall, the IR spectra indicate the successful synthesis of the Cu_2_(bdc)_2_(dabco) crystals, with the additional presence of amorphous bdc^2–^ containing regions and bands associated with Cu-bdc in the case of the LbL produced films. Further information on the nature of the crystalline constituents of the formed thin films will be obtained from X-ray diffractograms in the next section.

#### 3.2.2 Phase Analysis via X-ray Diffraction

The crystallographic phase analysis of both types of thin films is performed by comparing the experimental X-ray diffraction pattern with the calculated pattern based on the theoretically predicted crystal structure of Cu_2_(bdc)_2_(dabco). The 2D reciprocal space maps for both samples are presented in Figure 4. The left and right sides of the plots (positive and negative *q* axis) display identical GIXD patterns. Therefore, only one side is used for the phase analysis, while on the other side, the measured features are shown without being covered by the labeling.

**Figure 4:**
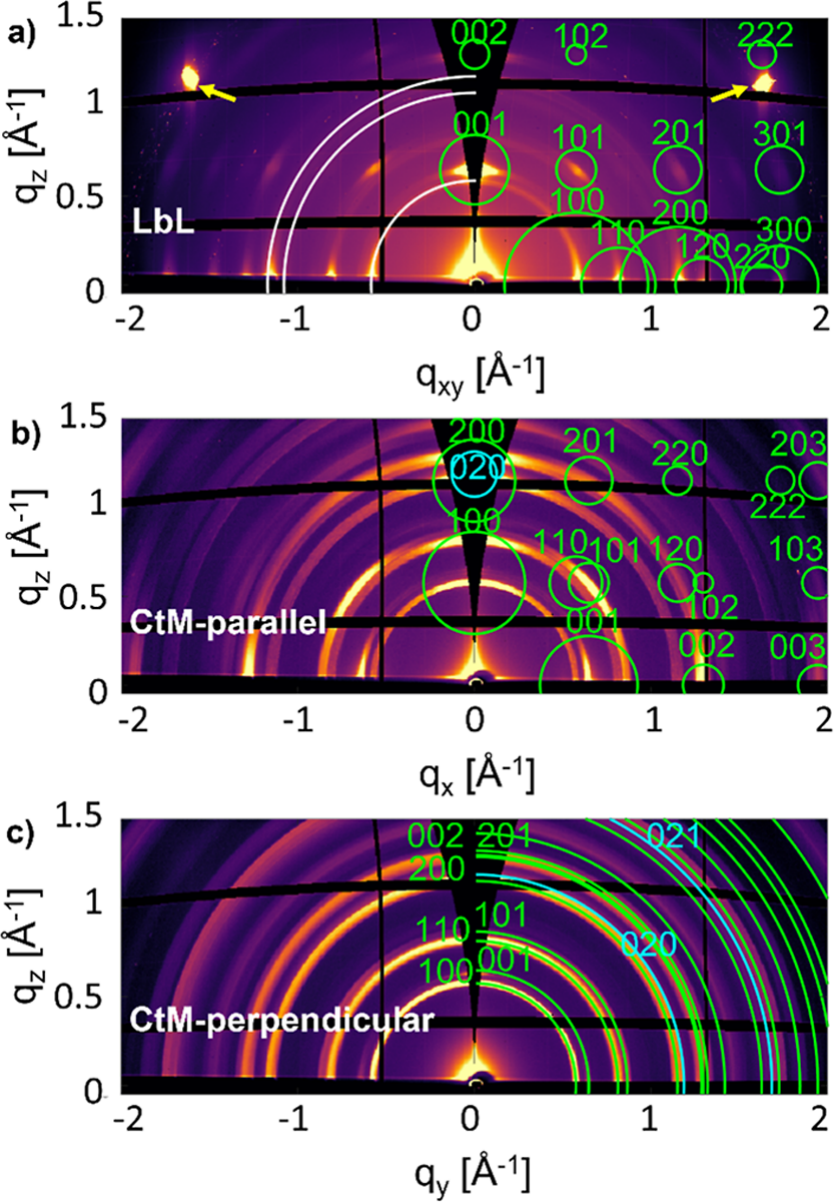
Reciprocal space maps of Cu_2_(bdc)_2_(dabco) prepared (a) by the LbL and (b,c) by the CtM methods. The maps are presented in (a) by integrated intensities for a full sample rotation, the two yellow arrows indicate 111 diffraction peaks from the silicon substrate. The maps (b,c) present specific cuts through reciprocal space (with the *x*-direction aligned along the preferred direction of the long axes of the nanobelts and the *y*-direction being perpendicular to it, see also main text). Calculated peak positions of Cu_2_(bdc)_2_(dabco), Cu-bdc, and of Cu­(OH)_2_ are given by quater circles in green, white, and cyan color, respectively. Small full circles indicate peak positions (via the centers of the circles) and intensities (via the areas of the circles scaled with the squares of the structure factors). Debye–Scherrer rings - plotted by quarter circles correspond to specific peak positions (a,c).

The sample grown by the LbL method shows distinct diffraction peaks that are found to be independent of the azimuthal orientation angle (denoted as ϕ) of the sample. This means that the rotating-GIXD data reveal that the GIXD patterns are indeed independent of the direction of the in-plane scattering vector. Therefore, the intensities for a complete sample rotation are summed, and the notation *q*_*xy*_ is used (Figure 4a).

The Bragg peaks appear at defined *q*_*xy*_/*q*_*z*_ positions, which can be unambiguously indexed based on the theoretically predicted crystal structure. The calculated 2D diffraction pattern is based on the assumption that the (001) plane of Cu_2_(bdc)_2_(dabco) crystals is parallel to the substrate surface; indeed the presence of defined peaks reveals a strong preferred orientation of the crystallites (see also discussion in Section 3.3). Peak positions as well as peak intensities fit well. Notably, the peaks are elongated into arc-type structures indicating an appreciable out-of-plane mosaicity.

For the phase analysis of the CtM sample, two reciprocal space maps are presented: one along the in-plane direction *q*_*x*_ (Figure 4b). Here, the *x*-direction is defined in the sample coordination system as being parallel to the preferred alignment direction of the long axes of the NBs. The second map is aligned along *q*_*y*_, i.e., within the sample surface, but perpendicular to *q*_*x*_ (Figure 4c). Again, it is the availability of the full rotating-GIXD data set that allows us to identify these two GIXD patterns as the ones most relevant for further analysis. In the first map (Figure 4b), diffraction maxima can be identified, although the arc-type structures are more pronounced than those in Figure 4a. Here, for plotting the diffraction maxima associated with the calculated 2D diffraction pattern, we assume a specific alignment of the crystallites with the Cu-bdc planes perpendicular to the plane of the substrate and the dabco linkers aligned along the *x*-direction. In contrast to the situation in Figure 4b, in Figure 4c exclusively Debye–Scherrer rings are observed, and the calculated diffraction patterns are shown by green quater circles. The *q*_*x*_/*q*_*z*_ peak positions in Figure 4b as well as the *q*-values of the Debye–Scherrer rings in Figure 4c can be well explained by the features derived from the theoretically predicted crystal structure of Cu_2_(bdc)_2_(dabco). Therefore, it is concluded that the same phase is present in both types of thin films (CtM and LbL), and that this phase is consistent with the calculated crystal structure.

To fully understand the structure of the CtM sample, the crystal structure of the NBs used during the thin film preparation process also must be taken into account. The Cu­(OH)_2_ NBs crystallize in the orthorhombic space group *Cmc*2_1_ with lattice constants of *a* = 2.947 Å, *b* = 10.593 Å, and *c* = 5.2564 Å.^94,95^ In the chosen representation of the diffraction data (*q*_*x*_ and *q*_*y*_ < 2 Å^–1^), the expected position of the 020 peak would overlap with that of Cu_2_(bdc)_2_(dabco) (Figure 4b) and the same would apply to the Debye–Scherrer ring at 1.684 Å^–1^ (Figure 4c). A more detailed analysis of the crystallographic properties of the Cu­(OH)_2_ substrate is given in Figures S7 and S8.

An identification of Cu-bdc, whose presence in the LbL film has been suggested by the IR spectra, is not straightforward. Nevertheless, despite the overlap in peak positions with Cu_2_(bdc)_2_(dabco), Cu-bdc could be identified by Debye–Scherrer rings located at *q* = 0.586 Å^–1^, at 1.172 Å^–1^, and at 1.080 Å^–1^, visualized by white circles in Figure 4a.^25^ No trace of Cu-bdc is found for the CtM sample, consistent with the IR data, indicating that by that growth technique, the pure Cu_2_(bdc)_2_(dabco) MOF was obtained. In this context it is also interesting to note that Brandner at al.^96^ reported the spontaneous transformation from Cu_2_(bdc)_2_(dabco) to Cu-bdc if a Cu_2_(bdc)_2_(dabco) film is exposed to humid conditions.

The excellent agreement of our experimental and theoretical results reveals that isostructural replacement is a versatile tool for generating guesses for possible crystal structures.^89^ In this context, it should, however, be mentioned that Cu_2_(bdc)_2_(dabco) is a particularly straightforward example and, thus, was also intentionally chosen for portraying the methodology described in the current manuscript. To our experience, in certain cases, the “search space” for starting structures for isostructural replacements needs to be broadened. Also, single-crystal diffraction data of the considered material can provide useful information, even though thin film structures often do not directly correspond to those found in the bulk due to variations in the growth conditions and due to the impact of the substrate triggering the growth of substrate-induced phases.^38,97^

### 3.3 Texture Determination via X-ray Diffraction

Knowing the crystal structure of Cu_2_(bdc)_2_(dabco) opens up the possibility of a thorough investigation of the preferred orientation of the crystallites within the thin films. To unambiguously determine the thin film texture, pole figures are used in combination with simulated stereograms based on the investigated crystal phase.^98^ A particular advantage of the rotating-GIXD method is that from the obtained data, pole figures for essentially any reflection can be calculated and compared to stereograms computed for the proposed crystal structure of Cu_2_(bdc)_2_(dabco). This allows the determination of the orientation distribution of the MOF crystallites and, with knowledge of the framework structure relative to the crystallographic lattice, the determination of the orientation of the bdc and dabco linkers.

#### 3.3.1 Layer-by-Layer Deposited Cu_2_(bdc)_2_(dabco)

The texture evaluation for the LbL sample was performed using the pole figures of the 001 Bragg peak and the 101 Bragg peak, with scattering vector lengths of *q* = 0.65 Å^–1^ and *q* = 0.86 Å^–1^, respectively. The pole figure of the 001 Bragg peak shows a clear peak in the center (Figure 5a), indicating that the (001) crystal plane is parallel to the substrate surface. This is consistent with analysis of the GIXD pattern (Figure 5a). In contrast, the pole figure of the 101 Bragg peak exhibits a circular shape (Figure 5b). Both pole figures are compared with the calculated stereogram (Figure 5c) of a single Cu_2_(bdc)_2_(dabco) crystal, assuming that it is aligned with the 001 pole perpendicular to the plane of the plot and the [100] direction parallel to its horizontal axis. Only the poles for the (001) plane and the {101} equivalent planes (namely, (101), (011), (−101), and (0–11)) are shown. The experimentally observed pole directions with high intensities (Figure 5a,b) agree with the calculated stereogram concerning their polar radii of ψ = 0° for the 001 and ψ = 41.7° for the 101 poles. However, the ring-like feature of the 101 pole figure (Figure 5b) is clearly different from the four individual directions of the poles corresponding to the (101), (011), (−101), and (0–11) planes present in the stereogram at defined polar angles (ϕ = 0°, 90°, 180°, and 270°). This implies that the thin film consists of crystallites with random azimuthal orientation relative to the normal of the substrate surface. This is expected,^99^ considering that the samples are grown on isotropic substrates. The fact that the crystals are aligned with the (001) planes parallel to the substrate but lack any in-plane alignment allows us to classify the distribution of the Cu_2_(bdc)_2_(dabco) crystals as uniplanarly textured.^60^ The Debye–Scherrer ring at *q* = 1.08 Å^–1^ associated with the Cu-bdc phase indicates that this phase displays random texture;^60^ a more detailed analysis of this unintended structure goes beyond the scope of the present paper.

**Figure 5:**
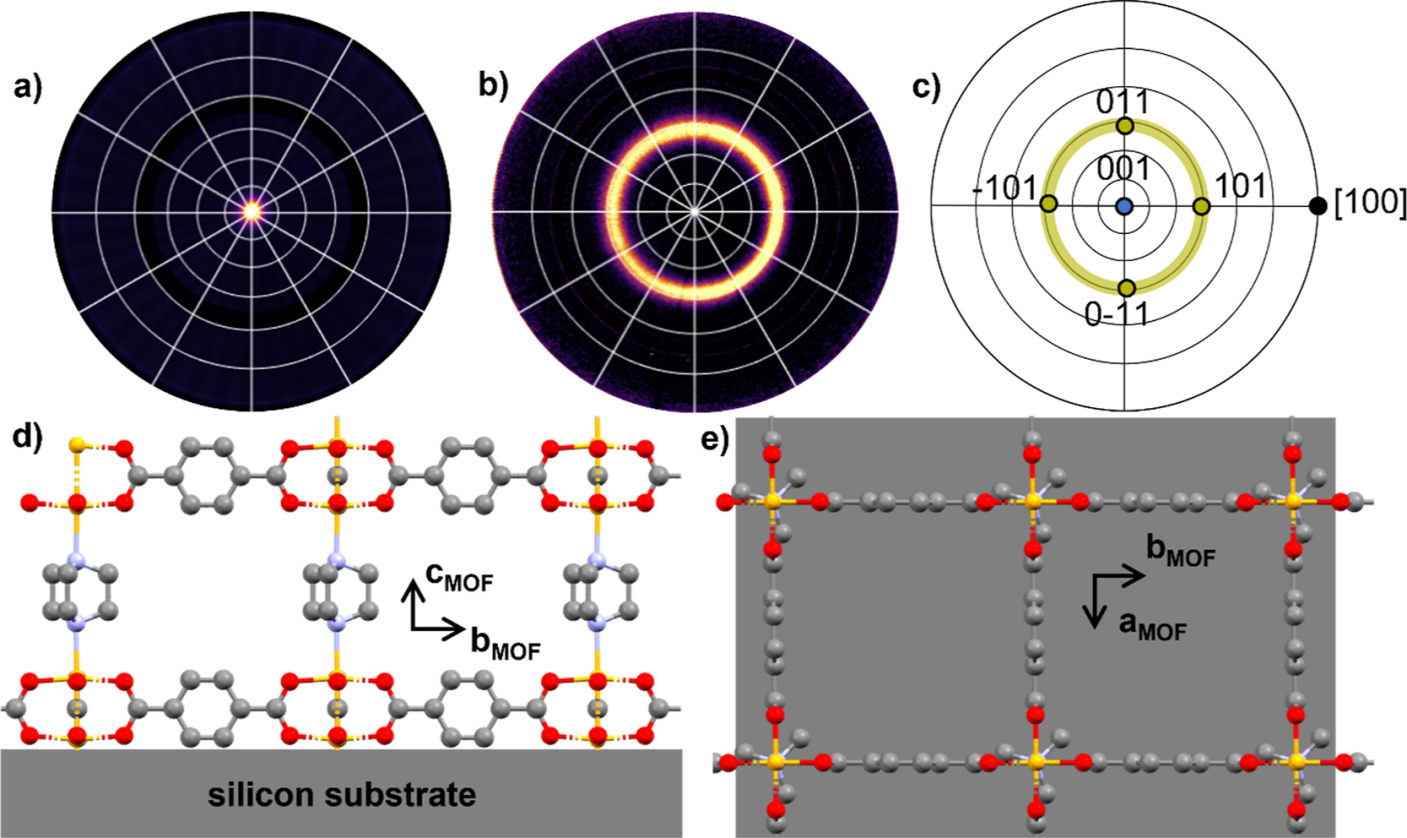
X-ray diffraction pole figures (a) of the 001 peak evaluated at *q* = 0.65 Å^–1^ and (b) of the 101 peak evaluated at *q* = 0.86 Å^–1^. Image (c) shows the calculated stereogram with the 001 pole in the center and the 101 poles at a tilt angle of ψ = 41.7°. The ring is characteristic for a uniplanar texture. The images (d,e) illustrate the extracted framework structure of Cu_2_(bdc)_2_(dabco) relative to the substrate surface in side and top view. The crystallographic axes indicate the crystal orientation of the MOF relative to the substrate surface. The brown, red, gray, and blue spheres show the copper, oxygen, carbon, and nitrogen atoms. The atomic arrangement is plotted with the visualization software Mercury from Cambridge Crystallographic Data Center.

Knowing the experimentally observed preferred orientation of the Cu_2_(bdc)_2_(dabco) crystals, the orientation of the linker molecules within the crystal structure can be identified, and the network structure of the MOF relative to the substrate surface can be determined. It is shown in a side view in Figure 5d and in a top view in Figure 5e: the dabco-linkers are oriented perpendicular to the substrate surface, while the bdc-linkers are arranged in a plane parallel to the substrate surface.

The uniplanar texture of the LbL film is associated with the absence of any in-plane order of the Cu_2_(bdc)_2_(dabco) crystallites; this is in agreement with the isotropic thin film microscope images. Disordered elongated plates with a lateral size between 200 and 600 nm and flakes with a size of about 1 μm are observed in good agreement with the results of McCarthy et al.^50^ The images are depicted in the Supporting Information, Figure S1a.

#### 3.3.2 Ceramic-to-MOF Grown Cu_2_(bdc)_2_(dabco)

The texture of the crystals in the CtM sample is also determined by using pole figures. Pole figures of the 100 Bragg peak (Figure 6a) and the 101 Bragg peak (Figure 6b) are generated with scattering vector lengths of *q* = 0.58 Å^–1^ and *q* = 0.86 Å^–1^, respectively. To obtain the orientation of the crystals, the pole figures are again compared with a calculated stereogram (Figure 6c). For the stereogram, a single crystal is aligned with the [001] direction parallel to the horizontal direction (i.e., *x* = axis) of the plot. The blue circles denoted as 0–10, 100, and 010 are obtained assuming the 100 poles of the Cu_2_(bdc)_2_(dabco) crystals perpendicular to the plane of projection (i.e., parallel to the *z*-axis). Provided that crystallites can rotate freely around the [001] axis, the poles of the {100} planes are distributed over multiple directions, as illustrated by the thick blue line in Figure 6c. In such a case, the [001] axis is referred to as the fiber axis. For the sake of comparison, Figure S10b shows the positions in stereographic projection calculated for specific degrees of rotation. Based on the positions of the poles of the {101} planes, one can estimate the positions of the respective poles for crystallites randomly rotated around the [001] axis. This is indicated by the yellow lines in Figure 6c. For illustrative purposes, the respective poles of one crystal, the one with the (100) plane parallel to the substrate surface, are represented by black circles. The calculated stereogram corresponds to an axial texture according to the classification of Heffelfinger and Burton.^60^

**Figure 6:**
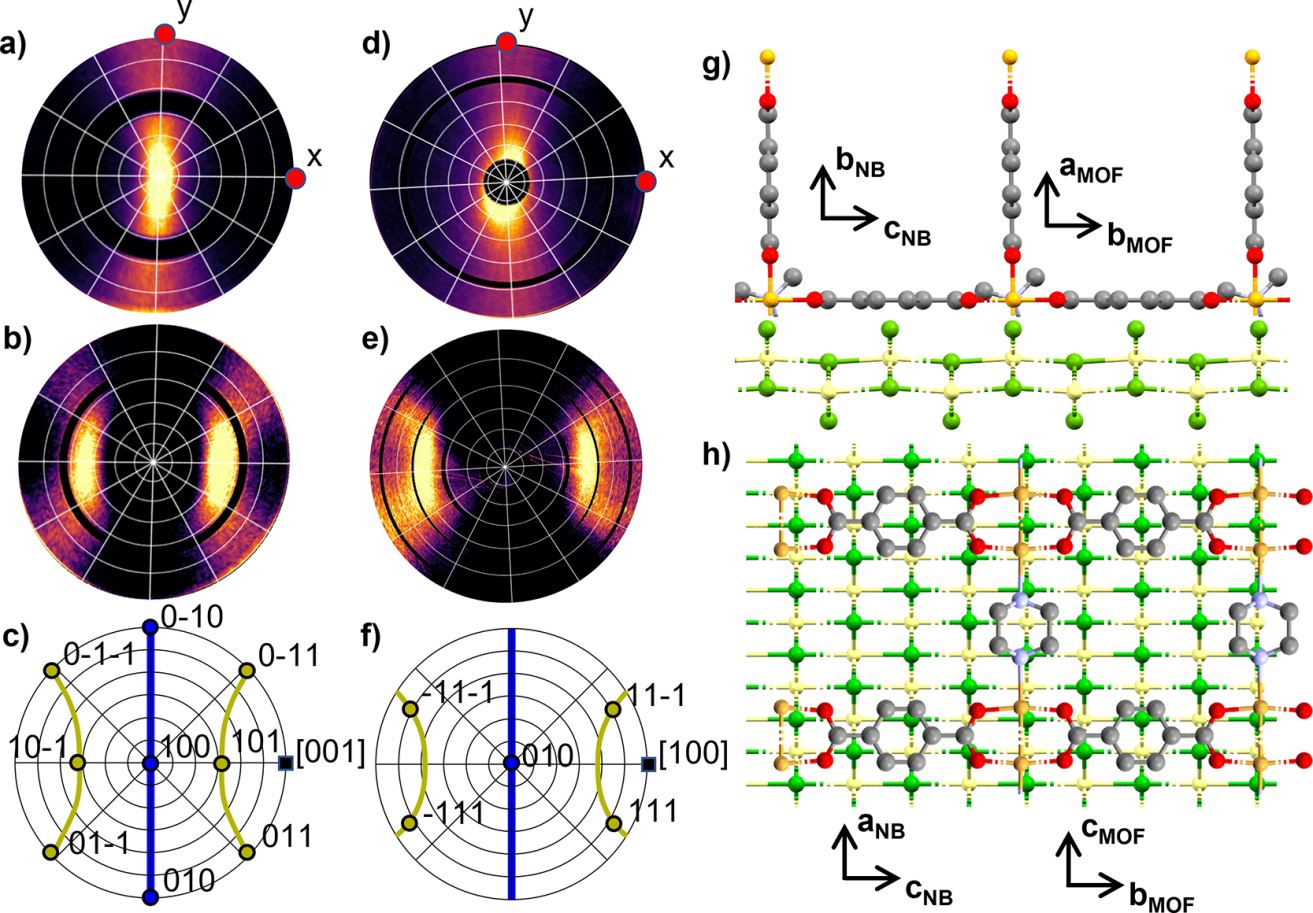
Panels (a,b) show X-ray diffraction pole figures of Cu_2_(bdc)_2_(dabco) of (a) the 100 peak taken at *q* = 0.58 Å^–1^, and (b) the 101 peak taken at *q* = 0.86 Å^–1^; the red dots give the *x* and *y* directions of the sample coordinate system. The black rings in the pole figures correspond to the blind spots of the detector. Panel (c) shows the stereogram for an ideal axial texture; the crystals are aligned with the [001] direction along the azimuthal fiber axis. Only the poles of the equivalent {100} and the {101} planes are plotted. Rotating the crystal around the [001] axis yields pole directions of {100} along the blue line and {101} poles along the yellow lines. Panels (d,e) show X-ray diffraction pole figures of Cu­(OH)_2_ nanobelts of (d) the 020 peak taken at *q* = 1.18 Å^–1^, (e) the 111 peak taken at *q* = 2.52 Å^–1^, and (f) the stereogram of an axial texture with [100] as unique direction, for (010) (blue) and for {111} equivalent poles (yellow). The rightmost panels illustrate the epitaxial order of Cu_2_(bdc)_2_(dabco) crystals at a (010) Cu­(OH)_2_ surface plotted in (g) side view and (h) top view. The green and yellow spheres show the oxygen atoms and the copper atoms of the Cu­(OH)_2_ nanobelt, respectively. The brown, red, gray and blue spheres show the copper, oxygen, carbon and nitrogen atoms of the MOF. The atomic arrangement is plotted with the visualization software Mercury from Cambridge Crystallographic Data Center.

The measured pole figures and the stereogram calculated for an ideal axial texture are correlated but do not match perfectly. First, the measured intensity distributions do not follow the theoretically estimated distributions (represented by the blue and yellow lines in the stereograms). They also show an angular spread (i.e., the features are broader than those ideally expected). Second, the observed intensities are not evenly distributed along the calculated directions of the stereogram. Both observations are related to a deviation of the alignment of the Cu_2_(bdc)_2_(dabco) crystals from the assumed equal distribution around the fiber axis.

The first deviation can be explained by a certain degree of misalignment of the [001] directions of the individual crystals relative to the ideal fiber axis. The angular spread of the [001] axes of the crystallites relative to the ideal fiber axis can be determined from the in-plane mosaicity. The corresponding ϕ-scan of the 100 Bragg peaks depicted in Figure S11 measures the in-plane mosaicity by the full width at half-maximum (fwhm) of the observed intensity curves. For Cu_2_(bdc)_2_(dabco) and for Cu­(OH)_2_, average values of 43.9° and 41.9° are found.

For the second mentioned deviation, the reason is a certain preference for the orientation of the crystallites with their (100) planes parallel to the substrate surface. A quantification of this observation by the out-of-plane mosaicity is difficult to perform since the 100 Bragg peak is not accessible for the entire range due to the blind spot of the detector. A clear experimental evidence for a preferred (100) orientation of the crystallites is, however, discussed in the context of Figure S12 in the Supporting Information. From the above arguments, it can be concluded that a considerable fraction of the crystallites is oriented with the (100) plane parallel to the substrates surface, which is a distinct deviation from an ideal axial texture.

A similar texture analysis can be performed on the crystalline Cu­(OH)_2_ NB precursor. The same data set from the rotating-GIXD experiment can be used since the X-ray diffraction features of the MOF and of the NBs are observed simultaneously, but at different *q*-values. Figure 6d and 6e shows the pole figures of Cu­(OH)_2_ taken at the 020 peak (*q* = 1.18 Å^–1^) and at the 111 peak (*q* = 2.52 Å^–1^). Figure 6f illustrates the calculated stereogram for Cu­(OH)_2_ crystals by assuming an ideal axial texture around the [100] direction as fiber axis. Comparison of the pole figures with the calculated stereogram again reveals clear similarities to but also deviations from an ideal axial texture. As in the case of Cu_2_(bdc)_2_(dabco) crystals, these deviations are caused by a non-ideal fiber texture arrangement of the Cu­(OH)_2_ crystallites. We consider this distinct deviation from an axial texture to be triggered by the plate-like geometry of the NBs,^25,52^ which favors the orientation of the large faces of the plates parallel to the substrate. In passing, we note that the texture of the NBs prior to the MOF growth is essentially the same as that of the remaining belts in the CtM films, as shown in Figures S7 and S8 for comparison.

The textures of Cu_2_(bdc)_2_(dabco) and of the Cu­(OH)_2_ NBs show two identical features: (i) the fiber axis preferentially points in identical directions and (ii) a specific crystallographic plane shows a preferred alignment parallel to the substrate surface. Comparing the out-of-plane mosaicities as well as the in-plane mosaicities reveals that the degree of disorder of the Cu_2_(bdc)_2_(dabco) crystallites is only slightly larger than those of the Cu­(OH)_2_ NBs (compare Figures S11 and S12). This suggests an epitaxial relationship between the Cu_2_(bdc)_2_(dabco) crystals and the Cu­(OH)_2_ NBs. In fact, the stereogram in Figure 6c,f reveals that [001]_MOF_ (the direction of the dabco molecules) is parallel to [100]_NBs_ (the long axis of the NBs). Moreover, from the preferred orientation of the crystals parallel to the substrate surface, it can be concluded that the {100} planes of the MOFs (i.e., (100)_MOF_ and (010)_MOF_) are preferably parallel to (010)_NB_. This means that these MOF planes, composed of bdc linkers in one direction and dabco linkers perpendicular to it, are parallel to the flat surface of the NBs.

As a consequence, one set of bdc-linkers is perpendicular to the flat Cu­(OH)_2_ NB surface (010 plane) (Figure 6g) and the second set of bdc-linkers, as well as the dabco-linkers, are parallel to it. The lattice mismatches *m*_1_ and *m*_2_ of the epitaxial thin film in both directions can be calculated according to^100^

$$\begin{array}{c} m_{1}=\frac{2c_{NB}-b_{MOF}}{b_{MOF}}\times 100\%\;\text{and}\\ m_{2}=\frac{{4a}_{NB}-c_{MOF}}{c_{MOF}}\times 100\% \end{array}\;$$

Two and four repeat units of the underlying Cu­(OH)_2_ substrate are covered by the bdc- and dabco-linkers, respectively; leading to commensurable lattices.^101^ Here, *c*_NB_ and *b*_MOF_ are the unit cell parameters along the short axis of the Cu­(OH)_2_ NBs and in the direction of a bdc-linker (Figure 6gh). The unit cell parameters *a*_NB_ and *c*_MOF_ are aligned in the direction of the long axis of the Cu­(OH)_2_ NBs and in the direction of the dabco linkers (Figure 6h). The resulting lattice mismatches amount to *m*_1_ ∼ 3% and m_2_ ∼ 8%, respectively. In this context it is worth noting that, values of mismatches below 10% are taken as tolerance for expecting heteroepitaxial growth of MOFs.^102^

The preferred alignment of the crystallites is in line with the experimentally observed fiber texture of the Cu_2_(bdc)_2_(dabco) crystallites discussed above.^25^ The thin film shows elongated structures aligned preferably in one direction with a characteristic length of about 3 μm and a width of 0.6 μm; the result is shown in the Supporting Information, Figure S1b. In this context, it is also worth noting that the orientation, homogeneity, and size of the MOF crystals in the CtM case strongly depend on the pursued synthesis protocol, the thickness of the sacrificial Cu­(OH)_2_ layer, the concentration of reagents, and the possible use of modulators, with all aspects offering ways for improving the film growth.^26,30,103^ For the sake of comparison, an optical microscopy image of the aligned Cu­(OH)_2_ NBs for a larger surface area is shown in the Supporting Information, Figure S9b.

## 4 Discussion

The solution of a crystal structure from a thin film is a difficult task, considering the comparably small number of detectable diffraction peaks. The presence of pronounced crystalline textures further complicates access to comprehensive diffraction data. Here, rotating-GIXD experiments (augmented by ab initio simulations) turn out to be a particularly useful tool, providing straightforward access to a detailed analysis of thin film textures and to diffraction peaks in the majority of reciprocal space. In contrast, GIXD experiments with static samples provides only a 2D cut through reciprocal space, which provides only sufficient information to characterize the orientation distribution of the crystallites only in specific cases (i.e., uniplanar or random textures).^60^

The potential of the rotating-GIXD approach is demonstrated by analyzing the crystallographic properties of Cu_2_(bdc)_2_(dabco) thin films. A model for the crystal structure of Cu_2_(bdc)_2_(dabco) is obtained based on the documented structure of Zn_2_(bdc)_2_(dabco) (depicted in Figure 1) by isostructural replacement.^89^ This is a particularly promising strategy for MOFs, as for this materials class, isomorphism has frequently been reported.^104–106^ In a subsequent step, the suggested geometry is optimized by DFT.^61^ The IR spectra calculated for that 3D Cu_2_(bdc)_2_(dabco) structure are consistent with the experimental data. For the LbL grown films, the measured IR data as well as XRD reveal additionally the presence of Cu-bdc crystallites (Figures 3 and 4a).

The calculated crystal structure is also found to be consistent with the X-ray diffraction experiments. In the case of the LbL sample, discrete Bragg peaks are observed in the GIXD patterns, which are independent of the azimuthal alignment of the sample (Figure 4a). These findings reveal that a uniplanar texture of the crystallites is present.^60^ This type of crystal orientation is frequently also denoted as “2D powder”.^107,108^ When calculating the peak pattern assuming a uniplanar texture with the (001) planes of the MOF crystals parallel to the substrate surface, an excellent agreement between the calculated and measured GIXD patterns is observed. For that texture, the direction of the dabco linker is perpendicular to the substrate surface, as depicted in Figure 5d.

A schematic picture of the orientation of the crystals relative to the substrate surface is given in Figure 7a.

**Figure 7:**
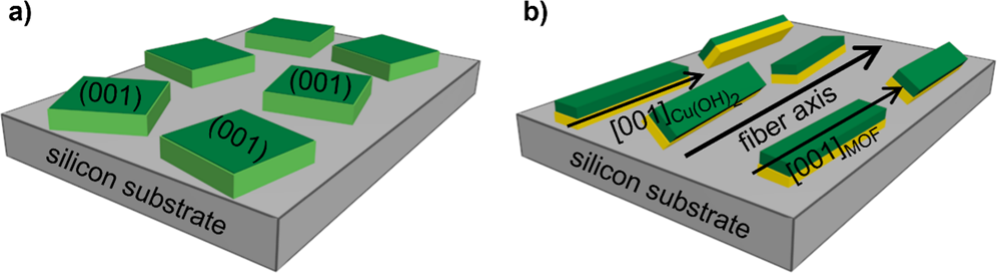
Schematic picture for the texture of Cu_2_(bdc)_2_(dabco) crystals within thin films. Panel (a) shows the uniplanar texture with the crystallographic (001) plane parallel to the substrate surface for the LbL grown film. Panel (b) illustrates the axial texture of the Cu_2_(bdc)_2_(dabco) crystals (green) epitaxially grown on the Cu­(OH)_2_ (yellow). The crystallographic [001] direction of the Cu_2_(bdc)_2_(dabco) crystals (corresponding to the axes of the dabco molecules) is aligned with the [100] direction of Cu­(OH)_2_ (the long axes of the nanobelts). Both axes are (close to) parallel to a fiber axis, which is directed along a specific azimuthal direction of the substrate surface defined during the deposition of the nanobelts. The planes of the nanobelts have a certain preference to be aligned parallel to the substrate surface, but nanobelts (and MOF crystals) with all possible rotations around the fiber axis are observed.

In the case of the CtM sample, the diffraction pattern is strongly dependent on the azimuthal alignment of the sample (Figure 6b,c). Peak patterns with arc-type structures as well as Debye–Scherrer rings appear. The observed anisotropy in an individual GIXD pattern would be consistent with films with preferred in-plane aligned crystallites or with samples with weak statistics.^109,110^ A full texture analysis is, however, not feasible based on a single GIXD image. Thus, it is necessary to collect GIXD patterns while systematically varying the azimuthal alignment of the sample (rotating-GIXD). This provides information on a large volume of the 3D reciprocal space, which can be presented in different ways.

For example, from the 3D reciprocal space mapping, pole figures can be calculated for the individual diffraction peaks. Plotting the peak intensities at a defined *q*-value as a function of the polar angles and radii (i.e., ϕ and ψ) yields pole figures, which are the basis of crystal texture analysis.^111^ In the case of the CtM sample, an axial texture together with a tendency to realize biaxially aligned crystals can be identified by comparing pole figures (Figure 6a,b) with calculated stereograms (Figure 6c). Additionally, the underlying Cu­(OH)_2_ NBs can be analyzed by pole figures, since they give rise to Bragg peaks at positions different from those arising from the Cu_2_(bdc)_2_(dabco) crystallites. Notably, for the Cu­(OH)_2_ NBs, a type of texture comparable with the one for the MOF crystallites is observed (Figures 6d–f and S8c–e). The similarity of the textures indicates that the texture of Cu_2_(bdc)_2_(dabco) is induced during film growth by the orientation of the Cu­(OH)_2_ NBs serving as sacrificial substrates. This is illustrated in Figure 7b.

The advantages of using rotating samples during the GIXD experiment are clearly apparent by the evaluation of the experimental data by pole figures. Still there are also clear limitations of the method. The peak collection strategy provides a common diffraction pattern of the thin film together with the substrate, e.g., in the case of the LbL sample, a peak stemming from the silicon substrate is also clearly visible (compare Figure 4a). In the present case, the separation of these two peak patterns can be easily performed, but it could be challenging in the case of low symmetry substrates (e.g., for muscovite substrates).^112^ It also should be mentioned that GIXD in general is limited by (i) specific areas where diffraction data are not accessible (compare Figure 4, e.g., along *q*_*z*_ at *q*_*xy*_ = 0 Å^–1^), (ii) diffraction peaks potentially having low resolution due to enhanced peak width, and by (iii) sparse diffraction patterns which are difficult to index. Notably, regarding the latter, significant progress has been made in recent years in the context of automatized indexing,^113^ sometimes also based on a machine learning-assisted peak assignment.

When trying to understand the origin of the fundamentally different textures of the two types of films, specific structural features of the substrate surface play a crucial role: The LbL sample is deposited on an oxidized silicon wafer, which forms an atomically flat but amorphous surface with isotropic character. Correspondingly, the two equivalent crystallographic directions of the MOF crystallites containing the bdc linkers (i.e., [100]_MOF_ and [010]_MOF_) do not display any preferred in-plane alignment (Figure 5c). The observation that the (001) plane of the crystallites is parallel to the SiO_2_ surface is tentatively attributed to the fact that such a texture is consistent with the formation of intact Cu-paddlewheels. Moreover, it is expected to maximize the van der Waals contact area between the substrate surface and the MOF. In the case of the CtM sample, an epitaxial alignment of the bdc and dabco linkers relative to the (010)_NB_ plane of the Cu­(OH)_2_ substrates is found (Figure 6g,h), with the bdc molecules aligned along [010]_NB_ and the dabco linkers aligned along [001]_NB_. Lattice mismatches between Cu_2_(bdc)_2_(dabco) and the Cu­(OH)_2_ substrates of 8% and 3% are observed, which is consistent with observations for other epitaxial MOF heterostructures.^102,114,115^ Both lattice mismatches still allow a favourable atomic arrangement of the MOF and the Cu­(OH)_2_ layers (Figure 6g,h).

As far as the impact of the different textures is concerned, it is interesting to mention that in the case of the LbL film, the extension of the pores parallel to the substrate surface is slightly larger than in the case of the CtM sample (compare Figure 5e with Figure 6h), which might have an impact on the diffusion of guest molecules through the films. Moreover, in a recent study, the use of polar apical linkers to generate gradients of the electrostatic energy has been suggested^116^ and (to some extent) also realized experimentally employing LbL growth.^117^ In such MOFs, the texture of the films determines the direction of the energy gradients. Moreover, when using optical chromophores as linkers, controlling the texture can be used to obtain polarized absorption and emission properties.^118,119^

## 5 Conclusions

This work shows, for the example of Cu_2_(bdc)_2_(dabco), how the crystal structure of thin film MOFs can be solved by starting from an isostructural compound (here: Zn_2_(bdc)_2_(dabco)) and subsequently combining theoretical simulation and experiments. To unambiguously confirm the structure suggested by isostructural replacement, IR spectroscopy as well as GIXD experiments are used. In the case of GIXD, it proves useful to map diffraction peaks in an as large as possible volume of reciprocal space. This can be achieved by the rotating-GIXD approach, recording GIXD patterns while rotating the thin film sample. This was done for two different types of Cu_2_(bdc)_2_(dabco) films, prepared either by an LbL approach or via CtM conversion starting from aligned Cu­(OH)_2_ NBs as substrates. To interpret the X-ray diffraction data for the thin films, certain textures of the films have to be assumed, which are later confirmed by pole figures directly extracted from the GIXD experiments. On the basis of these, identical crystal structures can be identified in both types of samples. Thin film preparation using the LbL technique results in a uniplanar texture with the (001) plane parallel to the substrate surface. In the case of CtM thin film preparation, a distorted axial texture is observed. In fact, crystals of the Cu_2_(bdc)_2_(dabco) MOF are found to grow epitaxially on the Cu­(OH)_2_ NBs with the epitaxial relations [001]_MOF_||[100]_NBs_ and {100}_MOF_||(010)_NBs_. These results show that in the two types of thin films, different alignments of the linker framework relative to the substrate surface are found. For the LbL grown films, the isotropic nature of the substrate surface is in accordance with the fact that both bdc-linkers are parallel to the substrate. When CtM conversion is employed, lattice matching between the MOF and the NB substrates leads to a fundamentally different texture with one bdc-linker and one dabco-linker parallel to the substrate surface. As its most important outcome, this work illustrates that GIXD with rotating substrates is a valuable tool for solving the crystal structure of thin films consisting of highly textured crystallites.

## Supplementary Material





## Data Availability

All original data can be accessed via repository of the Graz University of Technology (DOI 10.3217/87rsq-wey67).

## Notes

Additional details on thin film preparation and computational tests on geometry optimization; crystallographic characterization of pristine Cu­(OH)_2_ NBs; calculation on the magnetic order and IR spectra; and out-of-plane mosaicities of Cu­(OH)_2_ and the MOF (PDF)
Animations of the molecular vibrations (ZIP)
