# Supplementary material for: Identifying Structure and Texture of Metal–Organic Framework Cu2(bdc)2(dabco) Thin Films by Combining X‑ray Diffraction and Quantum Mechanical Modeling
Source: Cryst Growth Des. 2025 May 19;25(11):3665–79. doi: 10.1021/acs.cgd.4c01433 (PMC12151458; doi:10.1021/acs.cgd.4c01433)
Supplement: Supplementary file 1 [file cg4c01433_si_001.pdf]

Supporting Information

Identifying Structure and Texture of Metal-Organic Framework $Cu_2(bdc)_2(dabco)$ Thin Films by Combining X-ray Diffraction and Quantum Mechanical Modelling

Mario Fratschko,[a] Nina Strasser,[a] Narges Taghizade,[a] Mercedes Linares-Moreau,[b] Jan C. Fischer,[c] Tonghan Zhao,[c] Ian A. Howard,[c] Paolo Falcaro,[b] Egbert Zojer [a] and Roland Resel [a]

[a] Institute of Solid State Physics, Graz University of Technology, Petersgase 16, 8010 Graz, Austria

[b] Institute of Physical and Theoretical Chemistry, Graz University of Technology, Stremayrgasse 9, 8010 Graz, Austria

[c] Institute of Microstructure Technology, Karlsruhe Institute of Technology, Hermann-von Helmholtz-Platz 1, 76344 Eggenstein-Leopoldshafen, Germany

Emails: egbert.zojer@tugraz.at and roland.resel@tugraz.at,

CONTENT

The synthesis of the thin film samples used in this work is already described in literature. Therefore, the preparation procedure has been described in the main paper in a very compact way, but additional details are given together with the thin film morphology in chapter S1. A relevant part of the work relies on computational modelling. Therefore, a number of tests of the applied methodology were required for a reliable computation. They are provided in chapters S2.1 and S2.2, which also contain additional computational details. Information on the magnetic order and details of the calculation of the infrared spectra are provided in the chapters S2.3 and S2.4. More

detailed information about the experimentally observed infrared spectra are given in chapter S3. $Cu(OH)_2$ nanobelts were used as a specific precursor for preparation of $Cu_2(bdc)_2(dabco)$ by the ceramic-to-MOF (CtM) conversion. The crystalline properties of this type of substrate are discussed in chapter S4. The stereograms of the $Cu_2(bdc)_2(dabco)$ crystals and of the $Cu(OH)_2$ nanobelts are presented in detail in chapter S5. A comparison of the out-of-plane mosaicity of of $Cu_2(bdc)_2(dabco)$ and of $Cu(OH)_2$ nanobelts are given in chapter S6.

S1. Thin Film Preparation

S1.1 Layer-By-Layer Deposition

The procedure is carried out in accordance with the protocol established by McCarthy et al.[1] . In this approach, a conventional silicon substrate, which has been rinsed in ethanol, is transferred to an automated pump system substrate container at a temperature of 62°C. The system is then alternately soaked with a metal ion and a linker precursor solution. For the metal precursor solution, 1 mM of $Cu(CO_2CH_3)_2 \cdot H_2O$ was dissolved in ethanol by ultrasonication. Accordingly, for the linker precursor, 0.4 mM of 1,4-benzenedicarboxylic acid (H_2bdc) and 0.2 mM of 1,4-diazabicyclo[2.2.2]octane (dabco) were dissolved together in ethanol and were also ultrasonicated. At the beginning of the synthesis, the pump system was soaked with the metal precursor solution for 15 minutes, followed by two rinsing cycles with pure ethanol. The first of these rinsing cycles lasted for 0.5 minutes, while the second lasted for 4.5 minutes. After rinsing, the system was soaked for 30 minutes with the linker precursor solution, followed by the same rinsing procedure. The complete process was repeated 20 times with the ethanol rinsing cycles after exposure to the linker precursor solution as the last step. Subsequent to the last ethanol rinsing process, the samples were left to dry in laboratory atmosphere.

S1.2 Ceramic-to-MOF Conversion

The $Cu(OH)_2$ nanobelts were prepared according to the procedure reported in Falcaro et al.[2]. For the $Cu(OH)_2$ nanobelts (NBs), 3 mL of 0.15 M NH_4OH solution were first added dropwise to a 10 mL solution of 0.04 M $CuSO_4$-$5H_2O$ under constant stirring. After 5 min, 0.6 mL of 1.2 M NaOH was added dropwise. The solution was further stirred at room temperature for 1 h, and then heated

to 40˚C for 30 min without stirring. In the next step, the solution was centrifuged and washed alternatingly with water and ethanol, and the resulting wet powder (0.3 g) was dissolved in 10 mL of ethanol. Films of aligned nanobelts were deposited on silicon substrates according to a reported procedure.[3] The $Cu(OH)_2$ solution was injected with a syringe at a rate of 0.18 mL/min into a container (63 · 34 · 10 mm^3) filled with 13.8 mL of deionized water. An evenly distributed homogeneous film was formed on the water surface. Prior to use, a conventional silicon substrate with a native oxide layer was sonicated for 10 minutes in an acetone bath and later rinsed with ethanol and subsequently dried in a nitrogen stream. The cleaned silicon substrate was then pressed as parallel as possible onto the water surface and subsequently removed from there with a uniform motion, rinsed with ethanol and dried with nitrogen. $Cu_2(bdc)_2(dabco)$ MOF films were prepared by heteroepitaxial growth according to a reported procedure.[2,4] The nanobelt substrate was converted to $Cu_2(bdc)_2(dabco)$ by immersing the substrates for 1 h at 70 ˚C in a 10-mL methanol solution containing 6.64 mg of 1,4-benzenedicarboxylic acid (H_2bdc) and 287.1 mg of 1,4-diazabicyclo[2.2.2]octane (dabco).

S1.3 Thin Film Morphology

The morphology of the thin films was investigated using a JSM-6490LV scanning electron microscope (SEM) with an acceleration voltage of 10 kV. For the detection of the backscattered electrons a MP-44120 electron detector was used.

In both cases non-homogeneous coverages of the substrate surface with high surface roughness is observed. But there are also distinct differences in the morphology of the two types of films as shown in Figure S1. For the layer-by-layer prepared thin film elongated plates with a lateral size between 200 nm and 600 nm and flakes with a size of about 1 μm are observed. The ceramic-to-MOF thin film shows elongated structures aligned preferably in one direction with a characteristic length of about 3 μm and a width of 0.6 μm.

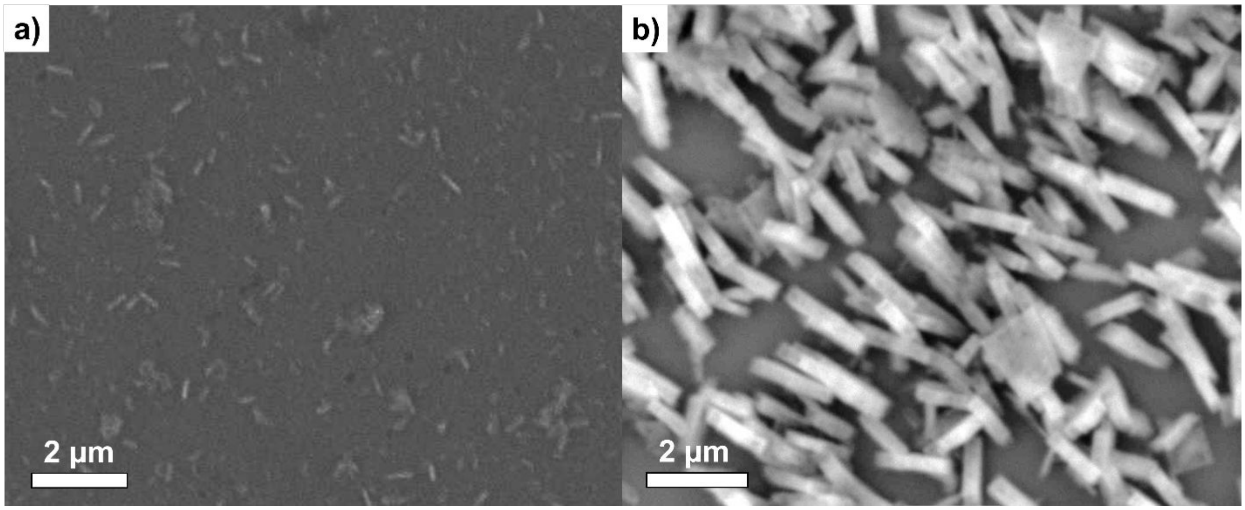

Figure S1. Scanning electron microscopy images of $Cu_2(bdc)_2(dabco)$ thin film crystallites grown by a) the layer-by-layer method and b) by the ceramic-to-MOF technique (in the latter case on axially aligned $Cu(OH)_2$ nanobelts). All films are deposited on native silicon oxide wafers.

S2. Computational Details

S2.1 Additional Computational Settings and Convergence Tests

To assess the impact of computational settings on the accuracy of our calculations, we conducted energy difference calculations for different setups using the Vienna ab initio simulation package[5] (VASP) and the Fritz Haber Institute ab initio molecular simulations package[6] (FHI-aims). Convergence is assumed to be achieved, when the change in total energy is less than 0.5 meV/atom from one setting to the next. For this converged setting it also turns out that the difference from the maximum setting is actually below 0.1 meV/atom. For the FHI-aims calculation, the typically well converged default "tight" basis set was used and reciprocal space was sampled using a converged 2×2×2 k grid (see Figure S2 (a)). The convergence criteria for the self-consistent field (SCF) procedure were set to the following values: 10^{-5} for the charge density, 10^{-6} eV for the total energy, 10^{-4} eV·Å^{-1} for the forces on atoms. To describe the occupation of the Kohn-Sham eigenstates, a Gaussian broadening function with default width of 0.01 eV was used. Relativistic effects were considered using the atomic Zeroth-Order Regular Approximation (ZORA).[7] Local

geometry optimizations were performed utilizing an enhanced version of the Broyden-Fletcher-Shanno-Goldfarb (BFGS) optimization algorithm, incorporating the trust radius method to efficiently locate minima on the potential energy surface.[6] A tolerance threshold of 10^{-3} eV·$Å^{-1}$ ensured satisfactory convergence during the optimization process.

In the case of VASP simulations, our investigation revealed that a 2×2×2 k-point grid[8] provided the required accuracy (see Figure S2 (b)), while employing an energy cutoff of 900 eV for the plane-wave basis yielded suitably converged results (see Figure S2 (c)). For the state occupation, we applied Gaussian smearing with the default width of 0.05 eV. The geometry relaxation procedure was carried out using the conjugate gradient algorithm[5,9] until the residual forces fell to below 10^{-3} eV·$Å^{-1}$. The projector augmented wave (PAW) files used in the simulations are specified in Table S1.

Table S1. The projector augmented wave (PAW) files used in our calculations, along with their unique headers. These headers provide the version and date of the PAW datasets, ensuring reproducibility and clarity in the computational setup.

Element	PAW File Header
C	PAW_PBE C 08Apr2002
Cu	PAW_PBE Cu 22Jun2005
H	PAW_PBE H 15Jun2001
N	PAW_PBE N 08Apr2002
O	PAW_PBE O 08Apr2002

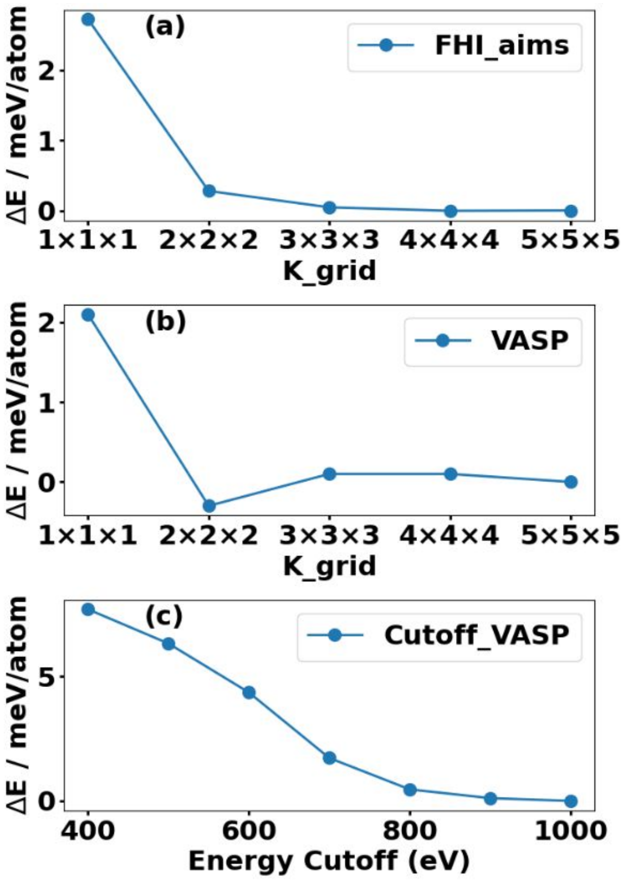

Figure S2. Energy difference (relative to the largest used setting) as a function of the k-grid in (a) and (b), and the plane wave energy cutoff in (c), calculated for crystalline $Cu_2(bdc)_2(dabco)$. The calculations were performed using the PBE functional with the DFT-D3 method with Becke-Johnson damping in VASP and the PBE functional with many-body dispersion (MBD_nl) correction in FHI-aims. (a) shows results from FHI-aims, while (b) and (c) present results obtained using VASP.

S2.2 Geometry Optimizations of $Zn_2(bdc)_2(dabco)$

As a first step, to benchmark the applied theoretical methodology the structure of $Zn_2(bdc)_2(dabco)$ was optimized starting from the *cif*-file reported in the Cambridge Crystallographic Data Centre.[10] Table S1 compares the simulation results for the unit cell (assuming P1 symmetry) to the experimentally reported structure. Considering that the simulations have been performed at de facto 0K (i.e., they disregard thermal expansion), the obtained agreement is clearly satisfactory.

Table S2. Lattice parameters and energy differences (relative to the minimum) for structure optimization of $Zn_2(bdc)_2(dabco)$) as obtained using the FHI-aims code.[10] The experimental data were measured at room temperature.

$Zn_2(bdc)_2(dabco)$	Cell-length [Å]	Cell-Angle [°]	ΔE [meV/unit cell]
No symmetry (*P1*) 0 K	a = 11.053 b = 11.053 c = 9.598	α = 89.772 β = 89.794 γ = 89.562	0.0
Orthogonal 0 K	a = 11.054 b = 11.053 c = 9.599	α = 90 β = 90 γ = 90	0.2
P4/mmm 298 K (CSD 1426627)[11]	a = 10.939 b = 10.939 c = 9.614	α = 90 β = 90 γ = 90	–

Notably, when switching to the Cu^{2+} ions, it is crucial to consider different spin configurations due to the presence of unpaired spins in the ions.

S2.3 Magnetic Configurations of $Cu_2(bdc)_2(dabco)$

Understanding magnetic interactions and predicting the behavior of magnetic materials is crucial in various scientific and technological applications. The Heisenberg-Dirac-Van Vleck Hamiltonian[12,13] provides a model to describe magnetism by capturing the interaction between the copper center.

The copper atoms in $Cu_2(bdc)_2(dabco)$ were analyzed under different spin configurations, including ferromagnetic (FM), antiferromagnetic (AFM), and non-magnetic (NM) states. The total magnetization of the unit cell, defined by the net difference between spin-up and spin-down electron densities, was set to zero Bohr magnetons (μ_B) for the AFM configuration and to 2.0 μ_B for the FM configuration. To realize the AFM state, the initial magnetic moments of the two copper atoms in each paddle wheel structure were aligned antiparallel. Conversely, for the FM state, the copper atoms in the primitive unit cell were initialized with their spins aligned in parallel. The optimized geometries for the different spin states are listed in Table 1 and the ground state of $Cu_2(bdc)_2(dabco)$ in both VASP and FHI-aims simulations is found to be antiferromagnetic. The results indicate that the AFM and FM states are quite close in energy, while the NM state is at significantly higher energy. To provide a more in-depth analysis of the results and to ensure the qualitatively correct nature of the calculated AFM and FM states, we calculated the spin densities and the local magnetic moments of the atoms, in analogy to the analysis presented in the MOF HKUST-1 for $Cu_2(bdc)_2(dabco)$[14].

In both, the FM and AFM configurations of $Cu_2(bdc)_2(dabco)$, the majority of the spin density is concentrated on the Cu atoms. In addition, a non-negligible spin density is observed on the oxygen atoms (O) in both states. The spin densities in both the FM and AFM states appear quite similar. The primary difference is that in the AFM state, the spin densities change signs between the two halves of the paddle wheels. This strongly suggests that the calculated open-shell singlet provides a reasonable representation of the AFM state. For a more detailed understanding of the local magnetic moments of the atoms, Table 2 summarizes the magnetic moments projected onto the individual atoms of the system. This yields the expected results.

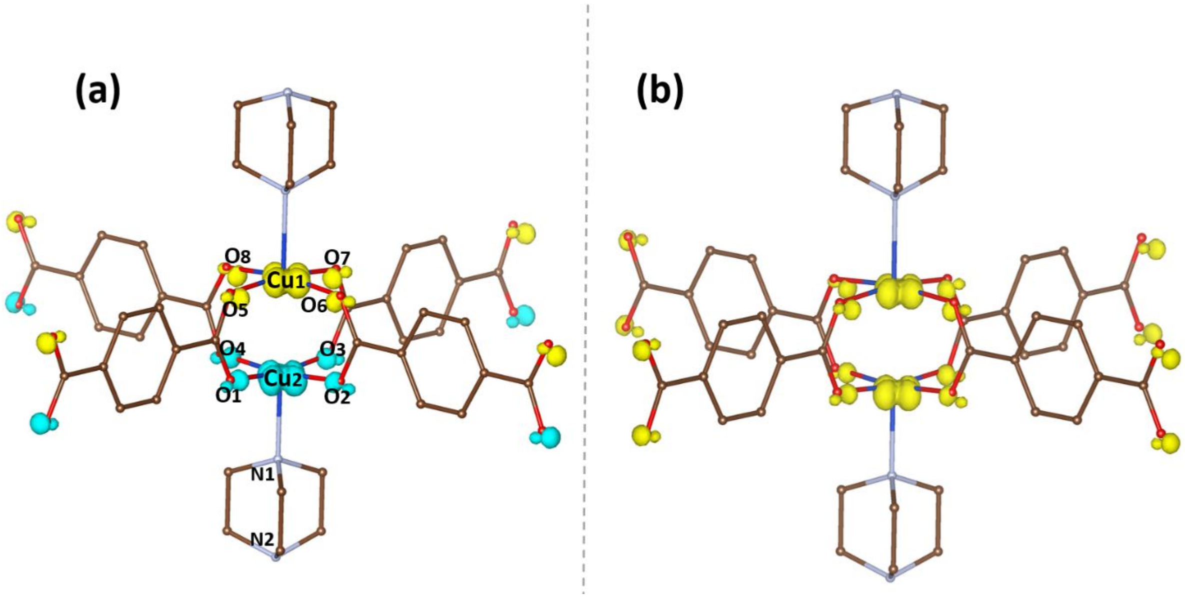

Figure S3. Spin densities of $Cu_2(bdc)_2(dabco)$ in the (a) antiferromagnetic and the (b) ferromagnetic configuration. The iso-surface level is set to ±0.2 electron /Å^3. Although the spin density results from both VASP and FHI-aims codes are consistent, the figure presented here has been specifically generated using the FHI-aims calculated spin densities. The figure was generated using VESTA version 3.5.8.[15]

Table S3. Local magnetic moment of $Cu_2(bdc)_2(dabco)$ for the ferromagnetic (FM) and antiferromagnetic (AFM) states calculated using VASP and FHI-aims. The magnetic moments of the atoms not listed in the table were either zero or close to zero.

VASP			FHI-aims		
	FM(μB)	AFM(μB)		FM(μB)	AFM(μB)
Cu_1	0.58	0.55	Cu_1	0.58	0.56
Cu_2	0.58	-0.55	Cu_2	0.58	-0.56
O_{1_4}	0.07	-0.07	O_{1_4}	0.09	-0.08
O_{5_8}	0.07	0.07	O_{5_8}	0.09	0.08
N_{1_2}	0.004	0.00	N_{1_2}	0.003	0.00

Furthermore, we investigated the bond lengths of $Cu_2(bdc)_2(dabco)$ for various magnetic configurations using both VASP and FHI-aims. Not surprisingly, the analysis of the results and the data in Table S3 show that the most significant changes in bond lengths upon switching between different magnetic states occur in the vicinity of the Cu atoms. Conversely, the bond

lengths between other atoms hardly change between different magnetic moments of the paddlewheels.

Table S4. Bond length of $Cu_2(bdc)_2(dabco)$ in different magnetic configurations of the paddle wheels calculated using the VASP and FHI-aims codes. The anti-ferromagenetic geometry has been considered for the further data evaluation.

VASP				FHI-aims			
atom-atom	NM (Å)	FM (Å)	AFM (Å)	atom-atom	NM (Å)	FM (Å)	AFM (Å)
Cu-Cu	2.598	2.548	2.588	Cu-Cu	2.596	2.560	2.578
Cu-N	2.245	2.236	2.262	Cu-N	2.239	2.259	2.260
Cu-O	2.004	2.013	1.998	Cu-O	2.034	1.995	1.992
C-C (dabco)	1.553	1.554	1.551	C-C (dabco)	1.553	1.551	1.551
C-C (benzene)	1.397	1.397	1.397	C-C (benzene)	1.397	1.397	1.397
C-N	1.480	1.479	1.481	C-N	1.479	1.481	1.481
C-H	1.088	1.088	1.088	C-H	1.088	1.088	1.088
C-O	1.273	1.275	1.275	C-O	1.269	1.271	1.271

S2.4 Infrared Spectra

As outlined in the main manuscript, accurately simulating the infrared (IR) spectra of MOFs requires the use of hybrid functionals, with B3LYP[16,17] being particularly useful. Due to the high computational cost, these calculations cannot be performed in VASP[18] and were instead carried out using CRYSTAL23[19]. In CRYSTAL23, the valence triple-zeta polarized (VTZP)[20] basis set was selected for the linker atoms (O, N, C, H), while the POB-TZVP-rev2[21] basis set, which is a revised version of a basis set series developed by Peintinger, Oliveira and Bredow, was used for Cu atoms. The VTZP basis set is a modified Ahlrichs-type basis set derived from the VTZ[20] basis set, which is an improved version of the TZV basis set that contains 11 s-type basis functions - one more than TZP[20] - along with a single d-type polarization function.

The shrinking factors for the diagonalization of the Hamiltonian matrix in reciprocal space for the Monkhorst-Pack net as well as for the Gilat net have no default value and were set to 1. The tolerances for the Coulomb and exchange series were set to the default values of 10^{-6}, 10^{-6}, 10^{-6}, 10^{-6} and 10^{-12}, respectively. Integrals with overlaps smaller than these thresholds were neglected or approximated. The DFT exchange-correlation term was evaluated on an XLGRID with 75 radial points and up to 974 points in the chemically relevant region. In the full geometry optimizations, the initial trust radius for the relaxation was set to 0.001 Bohr and the maximum trust radius was limited to 0.01 Bohr. The convergence criteria for the RMS of the gradient and of the displacement were set to $3 \cdot 10^{-5}$ Hartree/Bohr and to $4 \cdot 10^{-5}$ Bohr, respectively. The convergence threshold for the change of the total energy of the SCF cycle was set to 10^{-11} Hartree.

To demonstrate the consistence between VASP and CRYSTAL23 results, IR spectra predicted with both codes were compared using the Perdew-Burke-Ernzerhof[22] (PBE) functional with a 900 eV energy cutoff in VASP, as shown in Figure S3a. Additionally, the CRYSTAL23 results when employing the B3LYP functional are shown. The B3LYP hybrid functional particularly affects the strongly polar -COO- stretching vibrations by shifting them to higher wavenumbers. Figure S3b presents the frequency deviations between VASP and CRYSTAL23 for the PBE case, with the color of the dots representing deviations from the ideal case. Additionally, the root-mean-square deviation (RMSD) of frequencies was calculated to be 7.17 cm^{-1} over a range from 0 cm^{-1} to 1666 cm^{-1}, indicating a high degree of similarity between the frequencies obtained from VASP and CRYSTAL23[19]. Finally, Figure S3c illustrates the frequency deviations between the PBE and B3LYP functionals in CRYSTAL23. In this figure, all dots are colored blue, indicating that the frequencies calculated with PBE are consistently underestimated compared to those obtained with the hybrid functional. Overall, the root-mean square deviation (RMSD) between PBE and B3LYP calculations amounts to 34.8 cm^{-1}, which is not surprising, considering the fact that different functionals have been used.

To further validate the convergence of the VTZP basis set within CRYSTAL23, the basis set for the linker atoms was extended to include three polarization functions (two d-type and one f-type) from the Dunning-type cc-pVTZ[23] basis set, termed VTZPP. The IR spectra comparison using both

basis sets is presented in Figure S5. The VTZP basis set proved to be converged, as the comparison against VTZPP led to virtually no changes in the predicted IR spectrum, with an RMSD value of frequencies as low as 0.01 cm^{-1} over the frequency range from 0 to 1666 cm^{-1}.

An overview of all vibrational mode frequencies for $Cu_2(bdc)_2(dabco)$ within the compared experimental measurement range is provided in Table S4, along with corresponding animation files and an assignment of the vibration types. The animations were generated using Jmol[24] (version: 14.31.4).

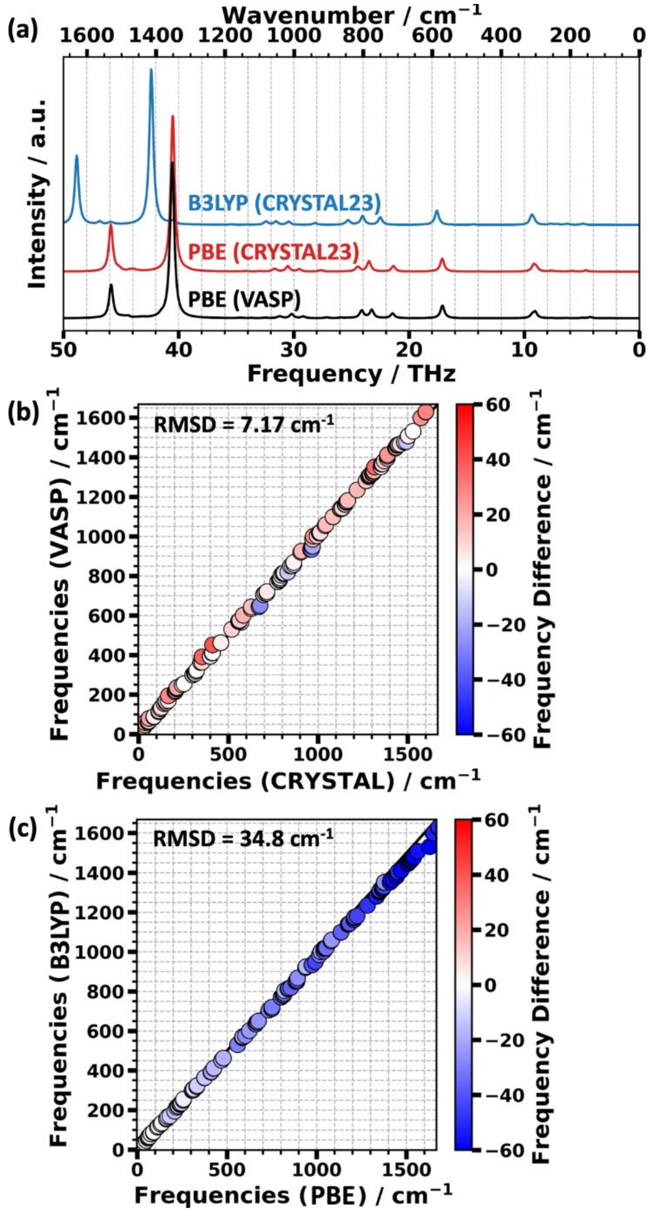

Figure S4. (a) Simulated IR spectra of $Cu_2(bdc)_2(dabco)$ using the PBE functional in CRYSTAL23 and VASP and also the hybrid functional B3LYP in CRYSTAL23 using the basis set VTZP for the linker atoms (O, N, C, H) and POB-TZVP-rev2 for Cu in the range from 0 cm^{-1} to 1666 cm^{-1}. (b) VASP calculated vs. CRYSTAL23 calculated frequencies of $Cu_2(bdc)_2(dabco)$ represented as dots. The PBE calculation with a 900 eV plane-wave energy cutoff is compared to the PBE calculation in CRYSTAL23 employing the basis set VTZP for the linker atoms and POB-TZVP-rev2 for the metal. The vibrations are displayed from 0 cm^{-1} to 1666 cm^{-1}. The dots are colored according to the frequency difference, with blue indicating an underestimation by CRYSTAL23 and red indicating an overestimation. The ideal case is shown as a straight black line. (c) PBE vs B3LYP calculated frequencies of $Cu_2(bdc)_2(dabco)$ using CRYSTAL23 with the basis set VTZP

for the linker atoms (O, N, C, H) and POB-TZVP-rev2 for Cu in the range from 0 cm^{-1} to 1666 cm^{-1}.

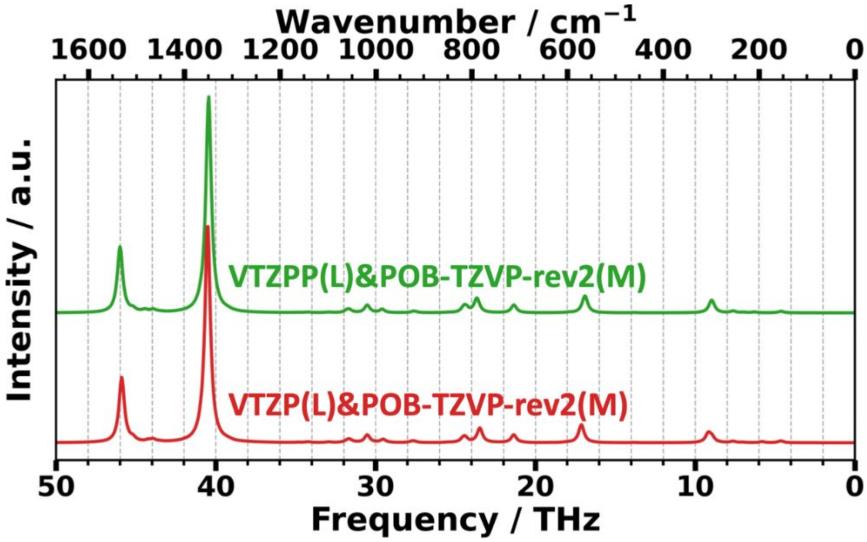

Figure S5. Simulated IR spectra of $Cu_2(bdc)_2(dabco)$ using the PBE functional in CRYSTAL23 with the basis set VTZP (bottom red curve) and VTZPP (top green curve) for the linker atoms (O, N, C, H) together with POB-TZVP-rev2 for Cu in both cases.

Table S5. Most relevant IR-active vibrational modes of $Cu_2(bdc)_2(dabco)$ calculated with CRYSTAL23 using the B3LYP functional together with the basis set VTZP for the linker and POB-TZVP-rev2 for the metal together with their assignments. The asterisk (*) indicates values that were obtained using the anharmonic correction. The classification of the vibrations is done by visual inspection and due to the complex structure of the studied system can only provide a limited characterization of the actual motions. The true displacements can be best assessed in the animations provided as additional Supporting Information.

Wavenumber [cm^{-1}]	IR intensity [KM/mol]	Name of animation file	Classification of vibration
1351	92.89	1351.mp4	Ring deformation mode
1414	2422.54	1414.mp4	Asymmetric backbone-stretching vibrations of the bdc linkers
1424	2540.81	1424.mp4	Asymmetric hydrogen scissoring of dabco
1444	17.98	1444.mp4	Asymmetric/symmetric backbone-deformation vibrations of the bdc linkers
1527	1.88	1527.mp4<br>1527a.mp4	-COO- stretching of bdc (on each paddlewheel in two opposing -COO groups the carbons move upwards, while in the two -COO groups perpendicular to them, they move downwards and vice versa such that no significant variation of the paddle-wheel dipole is expected; this is the reason for the very low IR intensity of the vibration. The vibration also comprises C-H scissoring vibrations in the dabco unit)
1529	33.16	1529.mp4	-C-H scissoring of dabco
1563	44.67	1563.mp4	Asymmetric -C-H bending of bdc
1629	2207.68	1629.mp4	Asymmetric phenyl deformation of bdc
3095 (2939*)	20.44	3095.mp4	-C-H stretching of dabco
3213 (3016*)	18.8	3213.mp4	-C-H stretching of bdc

S3. Experimental Infrared Spectra

Figure S5a shows the experimental IR spectra of the two distinct silicon substrates, across the entire accessible wavenumber range. The background for this measurement was collected in air. The figure shows, that at wavenumbers below ~1200 cm^{-1} there are numerous features that can be associated with IR absorption in the substrates. Absorption peaks at 512 cm^{-1}, 611 cm^{-1}, 738 cm^{-1}, 816 cm^{-1}, 888 cm^{-1} and 1110 cm^{-1} are various Si-R bands (R: H, O, CH_2, OH) from the silicon substrate as reported in the literature.[25–27] The absorption peak at 682 cm^{-1} corresponds to the Cu-O vibration from the $Cu(OH)_2$ nanobelts, which is only visible in the ceramic-to-MOF substrate.[3,28]

Figure S6b compares the experimental IR spectra for the $Cu_2(bdc)_2(dabco)$-MOF prepared by two distinct preparation methods and for Cu-bdc prepared *via* the ceramic-to-MOF technique across the entire accessible wavenumber range. The background for this measurement was recorded in air with an additional Si-substrate (to eliminate silicon vibrations from the measurement). It is worth to note, that the background measurement was performed only once with only one substrate for all measurements, which could be the reason for the observed substrate peaks below 1200 cm^{-1}. This shows, on the one hand, that there are no relevant spectral features at wavenumbers above those reported in the main paper. On the other hand, it illustrates that at wavenumbers below ~1200 cm^{-1} there are only absorption features from the substrate (see areas shaded in grey). The asterisk denotes an asymmetric COO vibration at 1577 cm^{-1}, indicative of Cu-bdc growth,[2] whereas the feature at 3568 cm^{-1} represents bands of hydroxyl ions.[28] In the case of the ceramic-to-MOF sample, the O-H adsorption peak is attributed to the underlying $Cu(OH)_2$ nanobelts substrate. In contrast, for the layer-by-layer sample, the additional Cu-bdc phase is responsible for the observed peak.

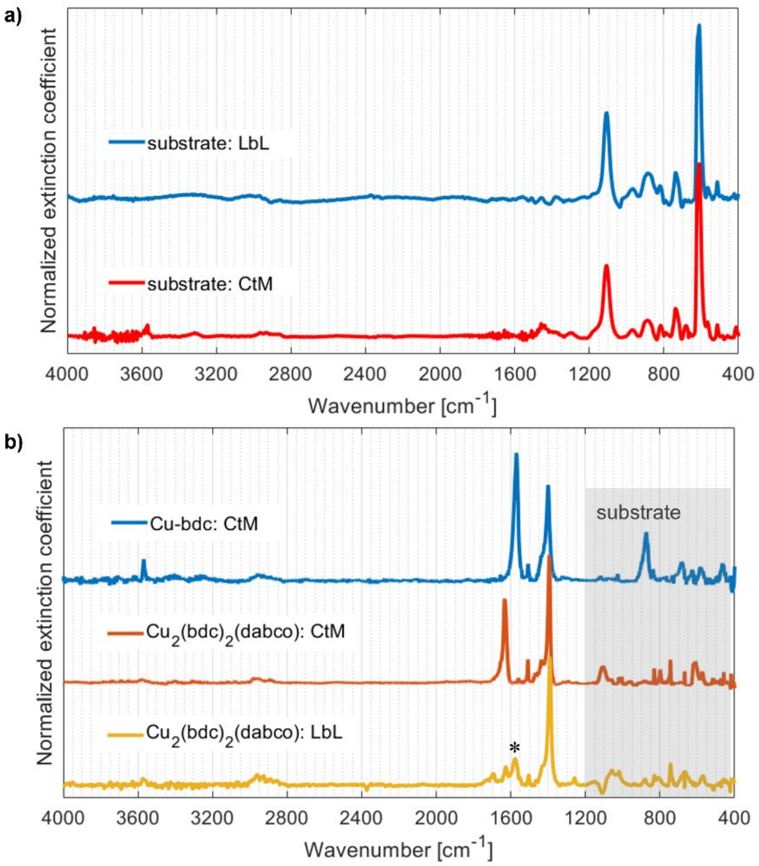

Figure S6. Experimental IR spectra of a) the substrates and b) the $Cu_2(bdc)_2(dabco)$ and Cu-bdc MOFs in the entire spectral range using both ceramic-to-MOF and layer-by-layer techniques. It is worth noting that the substrates were measured with air as a background, whereas the MOFs were measured with the Si substrate as a background. The infrared spectra of the used Si substrate is included to illustrate that below 1200 cm^{-1} no unambiguous distinction between MOF- and substrate-related features is possible. Notably, part of the nanobelts contained in the substrate are consumed in the CtM fabrication. The asterisk denotes the asymmetric COO^- vibration (1577 cm^{-1}) of Cu-bdc, indicative of its growth. The feature at 3568 cm^{-1} is assigned to bands of hydroxyl ions. In the ceramic-to-MOF sample, this O-H absorption peak originates from the $Cu(OH)_2$ nanobelts substrate, whereas in the layer-by-layer sample, it is a consequence of the additional Cu-bdc phase.

S4. Crystalline Properties of $Cu(OH)_2$ Nanobelts

A sample of $Cu(OH)_2$ nanobelts deposited on a silicon wafer was investigated by specular X-ray diffraction. In this approach, only out-of-plane information is collected, which, when combined

with GIXD (grazing incidence X-ray diffraction), provides access to significantly larger volumes of reciprocal space.

Specular X-ray diffraction was conducted with a PANalytical Empyrean diffractometer, with data collected exclusively from crystallographic planes that were parallel to the substrate surface. A sealed copper tube was employed in conjunction with parallel beam mirrors and a beam mask (10 mm) for monochromizing (λ = 1.5418 Å) and parallelizing the X-ray beam, respectively. The diffracted beam was detected with a PixCel3D detector operating as a 1D line detector. The data are represented in the reciprocal space by calculating $q = \frac{4\pi}{\lambda} sin\theta$.

The result is depicted in Figure S7, a comparison with a calculated powder pattern based on the known crystal structure of $Cu(OH)_2$ is given.[29,30] The observation of the specific series of Bragg peaks - namely *0kl* reflections - is a clear hint towards a fiber arrangement of the nanobelts with the [100] axis parallel to the substrate surface (see main paper Figure 7). To get more detailed information about the texture of the $Cu(OH)_2$ crystals, rotating grazing incidence X-ray diffraction (rotating-GIXD) was performed.

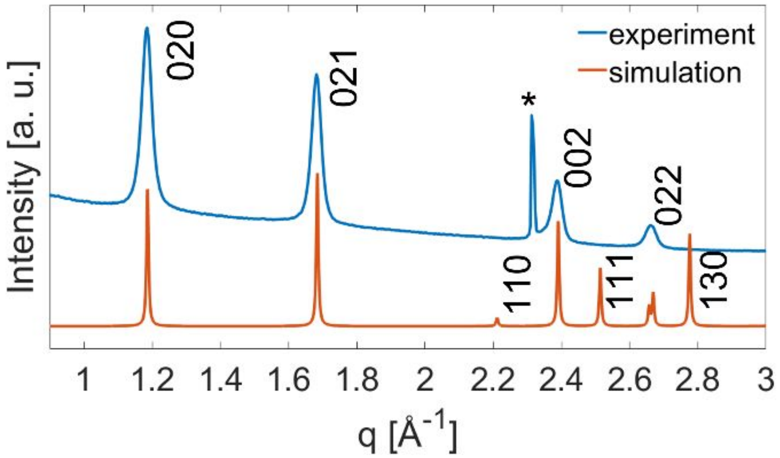

Figure S7. Specular X-ray diffraction of $Cu(OH)_2$ nanobelts deposited on silicon substrates together with indexation of the observed Bragg peaks. The simulation is based on randomly distributed crystallites. The asterisk (*) indicates the 200 Bragg peak of the Si substrate.[31]

Two reciprocal space maps for the $Cu(OH)_2$ nanobelts are presented in Figure S8a and S8b, representing GIXD measurements in q_x-direction and q_y-direction. The indexation is performed based on the known $Cu(OH)_2$ crystal structure[29,30]. In case of Figure S8a, supposedly prominent 020, 021, and 002 diffraction peaks are largely hidden due tor their appearance within the missing wedge of the GIXD experiments. Due to the large mosaicity of the crystallites, the associated features are still observable. Diffraction features are also clearly visible in Figure S8b, but as Debye-Scherrer rings. From the GIXD maps, pole figures are calculated for the 020 peak at a scattering vector q = 1.19 Å^{-1} and for the 111 peaks at q = 2.52 Å^{-1}. The results are depicted in Figure S8c and S8d. The pole figures can be compared with stereographic projections of $Cu(OH)_2$ crystals (Figure S8e). The observed distribution of the crystallites in both pole figures can be classified as an axial texture[32]. However, unlike for a fully axial texture as in the simulation (Figure S8e: blue line), the intensity distribution of the 020 poles along the "blue line" in the experiment is not homogeneous testifying to in a preferred crystal orientation with the (010) planes parallel to the substrate surface.

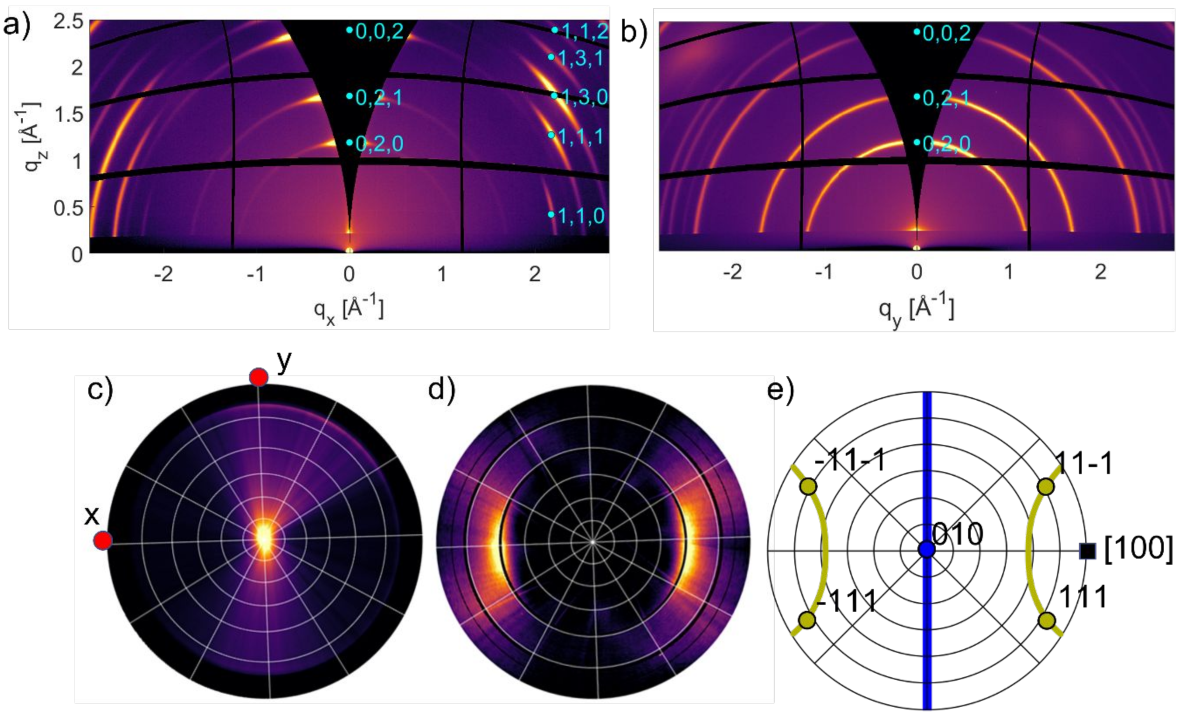

Figure S8. Reciprocal space maps of $Cu(OH)_2$ nanobelts deposited on a silicon wafer together with indexation of the observed Bragg peaks a) in q_x-direction and b) in q_y-direction. C) X-ray diffraction pole figures of the 020 Bragg peak taken at $q = 1.19$ Å^{-1} , the x- and y- direction of the sample coordinate system are given by discrete spots at the edge of the pole figure. d) pole figure of the 111 peak taken at $q = 2.52$ Å^{-1} . e) A stereogram of a $Cu(OH)_2$ crystallite with the pole of the (010) plane and the poles of the {111} planes (associated to the pole figures in c and d). Upon rotation around the [100] direction, the distribution of poles along the blue and yellow lines is observed.

The pole figure investigations reveal the orientation of the $Cu(OH)_2$ nanobelts relative to the substrate surface, which is sketched in Figure S9a. The crystallographic axes are related to the morphology of the nanobelts so that the long and short axes of the nanobelts are along the crystallographic a-axis and c-axis, respectively; the b-axis is peperpendicular to the flat surface of the nanobelt. An optical image of the nanobelts on the silicon substrate is provided in Figure S9b; the long axes of the nanobelts are observed as uniaxially aligned. Additional atomic force microscopy images of the $Cu(OH)_2$ nanobelts can be found in the literature.[3]

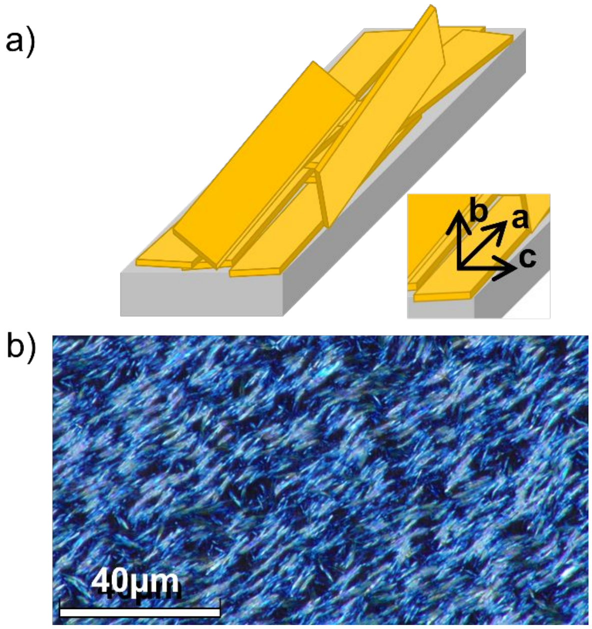

Figure S9. Arrangement of $Cu(OH)_2$ nanobelts at the substrate surface. a) Schematic image of the axial texture together with the crystallographic axes and b) optical microscopy image taken under crossed polarizers.

S5. Stereograms of $Cu(OH)_2$ and $Cu_2(bdc)_2(dabco)$

Figures 5c) and f) as well as Figure S8e give the stereograms of the uniaxially aligned crystallites only schematically by blue and yellow lines. For a comprehensive understanding we present the pole directions for individual crystal orientations within the fiber texture. A distribution of different crystal orientations is visualized in Figure S10 by stereograms for $Cu(OH)_2$ and for epitaxially grown $Cu_2(bdc)_2(dabco)$. Their crystal orientations are generated by rotation across the [100] axis of $Cu(OH)_2$ and across the [001] axis of $Cu_2(bdc)_2(dabco)$. Please note that both crystallographic directions ($[100]_{NB}$ and $[001]_{MOF}$) point in the same direction. The resulting poles of the selected crystal orientations are plotted with the same color (e.g. red, blue, grey, green, orange).

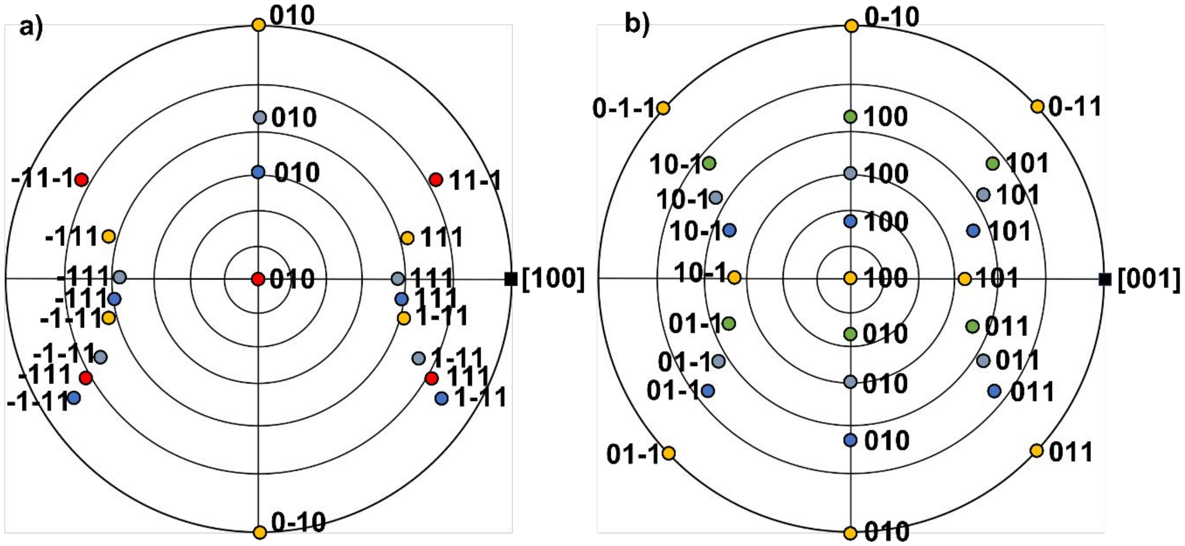

Figure S10. Stereograms with different crystal orientations a) of $Cu(OH)_2$ nanobelts and b) of $Cu_2(bdc)_2(dabco)$, in each of the two cases four crystal orientations are selected. Poles marked with the same color are related to each other: poles of the 010 and {111} planes for $Cu(OH)_2$ and poles of the {100} and {101} planes for $Cu_2(bdc)_2(dabco)$ are depicted. Black squares denote the crystallographic directions ([100] for $Cu(OH)_2$ and [001] for $Cu_2(bdc)_2(dabco)$) which coincide with the fiber axis of the underlying axial texture.

S6. Mosaicities of $Cu(OH)_2$ and $Cu_2(bdc)_2(dabco)$ Crystals

Deviations from the ideal fiber texture are expressed by the mosaicities of the investigated crystals. Two different directions of the mosaicity are considered: in-plane mosaicity and out-of-plane mosaicity. Both types of mosaicities are determined for each of the of the two types of fiber textured crystals, namely $Cu(OH)_2$ and $Cu_2(bdc)_2(dabco)$.

S6.1 In-plane Mosaicities

The in-plane mosaicity is taken from our rotating-GIXD data by plotting the intensity distribution of a selected Bragg peak along the ϕ - axis for a complete sample rotation. The 100 Bragg peak is chosen for $Cu_2(bdc)_2(dabco)$ and the 020 Bragg peak for $Cu(OH)_2$. The results are given in Figure S11. The in-plane mosaicity is represented by the full width at half maximum (FWHM) of the observed intensity distributions. Values of 46.7° and 41.1° are found for $Cu_2(bdc)_2(dabco)$ and 45.2° and 38.7° for $Cu(OH)_2$.

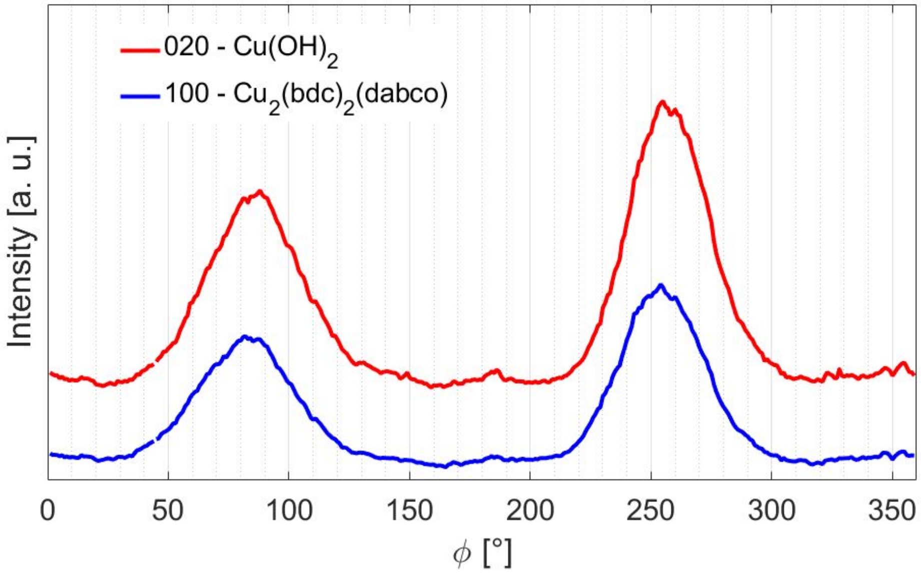

Figure S11. Intensity distribution of single Bragg peaks along a full sample rotation around the surface normal. The 100 Bragg peak is chosen for $Cu_2(bdc)_2(dabco)$ and the 020 peak for $Cu(OH)_2$.

S6.2 Out-of-plane Mosaicities

In case of the out-of-plane mosaicity two different cuts through the reciprocal space have to be considered: one cut along the x,z - plane and a second one along the y,z - plane. These mosaicities

are taken from the peak intensities along the Debye-Scherrer rings. The 100 Bragg peak is chosen for $Cu_2(bdc)_2(dabco)$ and the 020 Bragg peak for $Cu(OH)_2$. The intensities are plotted as a function of the polar angle ψ, with ψ between 90° and -90°. The results are shown in Figure S12. Due to the unfortunate choice of the detector position which placed the 020 peak of $Cu(OH)_2$ close to an blind spots of the detector (compare Figure 3), the results of an equivalent sample (measured with another sample detector distance) are depicted.

In case of the out-of-plane mosaicity in the *x,y* - plane a defined peak as well as a constant intensity is observed. The constant intensity refers to an ideal axial texture, while the peak at ψ = 0° arises due to preferred orientation of the crystallites - (100) for $Cu_2(bdc)_2(dabco)$ and (020) of $Cu(OH)_2$ - parallel to the substrate surface.

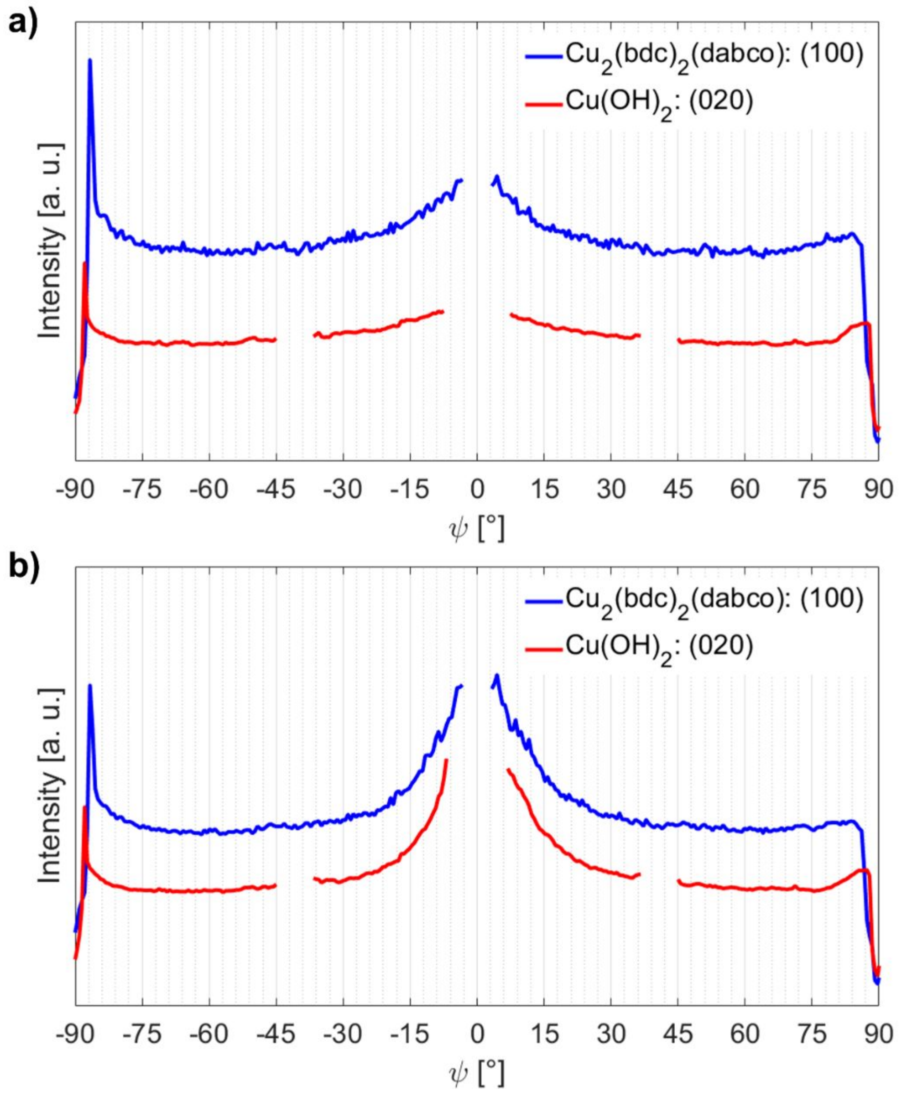

Figure S12. Intensity profile plotted along the radial angle ψ for the 100 and 200 Bragg peaks of $Cu_2(bdc)_2(dabco)$ and $Cu(OH)_2$, respectively. a) Plot across the *x,z* - plane and b) plot across the *y,z* - plane. The open parts of the curves are due to blind spots of the detector or inaccessible regions of reciprocal space due to the measurement geometry; the intensity spikes slightly below 90˚ and above -90˚ are due to Yoneda peaks.

REFERENCES

(1) McCarthy, B. D.; Liseev, T.; Beiler, A. M.; Materna, K. L.; Ott, S. Facile Orientational Control of M2L2P SURMOFs on ⟨100⟩ Silicon Substrates and Growth Mechanism Insights for Defective MOFs. *ACS Appl. Mater. Interfaces* **2019**, *11* (41), 38294–38302. https://doi.org/10.1021/acsami.9b12407.

(2) Falcaro, P.; Okada, K.; Hara, T.; Ikigaki, K.; Tokudome, Y.; Thornton, A. W.; Hill, A. J.; Williams, T.; Doonan, C.; Takahashi, M. Centimetre-Scale Micropore Alignment in Oriented Polycrystalline Metal–Organic Framework Films via Heteroepitaxial Growth. *Nature Mater* **2017**, *16* (3), 342–348. https://doi.org/10.1038/nmat4815.
(3) Linares-Moreau, M.; Brandner, L. A.; Kamencek, T.; Klokic, S.; Carraro, F.; Okada, K.; Takahashi, M.; Zojer, E.; Doonan, C. J.; Falcaro, P. Semi-Automatic Deposition of Oriented Cu(OH)2 Nanobelts for the Heteroepitaxial Growth of Metal–Organic Framework Films. *Advanced Materials Interfaces* **2021**, *8* (21), 2101039. https://doi.org/10.1002/admi.202101039.
(4) Okada, K.; Nakanishi, M.; Ikigaki, K.; Tokudome, Y.; Falcaro, P.; J. Doonan, C.; Takahashi, M. Controlling the Alignment of 1D Nanochannel Arrays in Oriented Metal–Organic Framework Films for Host–Guest Materials Design. *Chemical Science* **2020**, *11* (30), 8005–8012. https://doi.org/10.1039/D0SC02958K.
(5) Kresse, G.; Furthmüller, J. Efficiency of Ab-Initio Total Energy Calculations for Metals and Semiconductors Using a Plane-Wave Basis Set. *Computational materials science* **1996**, *6* (1), 15–50.
(6) Blum, V.; Gehrke, R.; Hanke, F.; Havu, P.; Havu, V.; Ren, X.; Reuter, K.; Scheffler, M. Ab Initio Molecular Simulations with Numeric Atom-Centered Orbitals. *Computer Physics Communications* **2009**, *180* (11), 2175–2196. https://doi.org/10.1016/j.cpc.2009.06.022.
(7) Lenthe, E. van; Baerends, E. J.; Snijders, J. G. Relativistic Regular Two-component Hamiltonians. *The Journal of Chemical Physics* **1993**, *99* (6), 4597–4610. https://doi.org/10.1063/1.466059.
(8) VASP. KPIONT. https://www.vasp.at/wiki/index.php/KPOINTS (accessed 2024-06-28).
(9) Kresse, G.; Joubert, D. From Ultrasoft Pseudopotentials to the Projector Augmented-Wave Method. *Physical review b* **1999**, *59* (3), 1758.
(10) Kim, Y.; Haldar, R.; Kim, H.; Koo, J.; Kim, K. The Guest-Dependent Thermal Response of the Flexible MOF Zn 2 (BDC) 2 (DABCO). *Dalton Transactions* **2016**, *45* (10), 4187–4192. https://doi.org/10.1039/C5DT03710G.
(11) Kim, Y.; Haldar, R.; Kim, H.; Koo, J.; Kim, K. The Guest-Dependent Thermal Response of the Flexible MOF Zn 2 (BDC) 2 (DABCO). *Dalton Transactions* **2016**, *45* (10), 4187–4192.
(12) Van Vleck, J. H. A Survey of the Theory of Ferromagnetism. *Reviews of Modern Physics* **1945**, *17* (1), 27.
(13) Becke, A. D. Density-functional Thermochemistry. I. The Effect of the Exchange-only Gradient Correction. *The Journal of chemical physics* **1992**, *96* (3), 2155–2160.
(14) Strasser, N.; Wieser, S.; Zojer, E. Predicting Spin-Dependent Phonon Band Structures of HKUST-1 Using Density Functional Theory and Machine-Learned Interatomic Potentials. *International Journal of Molecular Sciences* **2024**, *25* (5), 3023.
(15) Momma, K.; Izumi, F. VESTA: A Three-Dimensional Visualization System for Electronic and Structural Analysis. *Journal of Applied Crystallography*, 2008, *41*, 653–658.
(16) Becke, A. D. Density-Functional Thermochemistry. III. The Role of Exact Exchange. *The Journal of Chemical Physics* **1993**, *98* (7), 5648–5652. https://doi.org/10.1063/1.464913.
(17) Lee, C.; Yang, W.; Parr, R. G. Development of the Colle-Salvetti Correlation-Energy Formula into a Functional of the Electron Density. *Phys. Rev. B* **1988**, *37* (2), 785–789. https://doi.org/10.1103/PhysRevB.37.785.
(18) Kresse, G.; Furthmüller, J. Efficiency of Ab-Initio Total Energy Calculations for Metals and Semiconductors Using a Plane-Wave Basis Set. *Computational Materials Science* **1996**, *6* (1), 15–50. https://doi.org/10.1016/0927-0256(96)00008-0.

(19) Erba, A.; Desmarais, J. K.; Casassa, S.; Civalleri, B.; Donà, L.; Bush, I. J.; Searle, B.; Maschio, L.; Edith-Daga, L.; Cossard, A.; Ribaldone, C.; Ascrizzi, E.; Marana, N. L.; Flament, J.-P.; Kirtman, B. CRYSTAL23: A Program for Computational Solid State Physics and Chemistry. *J. Chem. Theory Comput.* **2022**, acs.jctc.2c00958. https://doi.org/10.1021/acs.jctc.2c00958.

(20) Schäfer, A.; Horn, H.; Ahlrichs, R. Fully Optimized Contracted Gaussian Basis Sets for Atoms Li to Kr. *The Journal of Chemical Physics* **1992**, *97* (4), 2571–2577. https://doi.org/10.1063/1.463096.

(21) Vilela Oliveira, D.; Laun, J.; Peintinger, M. F.; Bredow, T. BSSE-Correction Scheme for Consistent Gaussian Basis Sets of Double- and Triple-Zeta Valence with Polarization Quality for Solid-State Calculations. *Journal of Computational Chemistry* **2019**, *40* (27), 2364–2376. https://doi.org/10.1002/jcc.26013.

(22) Perdew, J. P.; Burke, K.; Wang, Y. Generalized Gradient Approximation for the Exchange-Correlation Hole of a Many-Electron System. *Phys. Rev. B* **1996**, *54* (23), 16533–16539. https://doi.org/10.1103/PhysRevB.54.16533.

(23) Dunning, T. H. Gaussian Basis Sets for Use in Correlated Molecular Calculations. I. The Atoms Boron through Neon and Hydrogen. *The Journal of Chemical Physics* **1989**, *90* (2), 1007–1023. https://doi.org/10.1063/1.456153.

(24) Jmol: An Open-Source Java Viewer for Chemical Structures in 3D. http://www.jmol.org/.

(25) Rokita, M.; Handke, M.; Mozgawa, W. Spectroscopic Studies of Polymorphs of AlPO4 and SiO2. *Journal of Molecular Structure* **1998**, *450* (1), 213–217. https://doi.org/10.1016/S0022-2860(98)00430-X.

(26) Warntjes, M.; Vieillard, C.; Ozanam, F.; Chazalviel, J.-N. Electrochemical Methoxylation of Porous Silicon Surface. *J. Electrochem. Soc.* **1995**, *142* (12), 4138. https://doi.org/10.1149/1.2048476.

(27) Kunst, S.; Beltrami, L.; Cardoso, H.; Santana, J.; Sarmento, victor hugo; Muller, I.; Malfatti, C. F. Characterization of Siloxane-Poly(Methyl Methacrylate) Hybrid Films Obtained on a Tinplate Substrate Modified by the Addition of Organic and Inorganic Acids. *Materials Research* **2015**, *18*, 151–163. https://doi.org/10.1590/1516-1439.299514.

(28) Park, S.-H.; Kim, H. J. Unidirectionally Aligned Copper Hydroxide Crystalline Nanorods from Two-Dimensional Copper Hydroxy Nitrate. *J. Am. Chem. Soc.* **2004**, *126* (44), 14368–14369. https://doi.org/10.1021/ja047425w.

(29) von Jaggi, H.; Oswald, H. R. Die Kristallstruktur Des Kupferhydroxids Cu(OH)2. *Acta Cryst* **1961**, *14* (10), 1041–1045. https://doi.org/10.1107/S0365110X61003016.

(30) Oswald, H. R.; Reller, A.; Schmalle, H. W.; Dubler, E. Structure of Copper(II) Hydroxide, Cu(OH)2. *Acta Cryst C* **1990**, *46* (12), 2279–2284. https://doi.org/10.1107/S0108270190006230.

(31) Zaumseil, P. High-Resolution Characterization of the Forbidden Si 200 and Si 222 Reflections. *J Appl Crystallogr* **2015**, *48* (2), 528–532. https://doi.org/10.1107/S1600576715004732.

(32) Heffelfinger, C. J.; Burton, R. L. X-Ray Determination of the Crystallite Orientation Distributions of Polyethylene Terephthalate Films. *Journal of Polymer Science* **1960**, *47* (149), 289–306. https://doi.org/10.1002/pol.1960.1204714926.
